# Determination of the Morphometric Characteristics of Larval Instars in the Sap Beetle *Urophorus humeralis* (Coleoptera: Nitidulidae)

**DOI:** 10.3390/insects17030344

**Published:** 2026-03-21

**Authors:** Kang Chang, Yilin Guo, Youssef Dewer, Xiaoxiao Chen, Suqin Shang

**Affiliations:** 1 Biocontrol Engineering Laboratory of Crop Diseases and Pests of Gansu Province, College of Plant Protection, Gansu Agricultural University, Lanzhou 730070, China; 15294346907@163.com (K.C.);; 2 Central Agricultural Pesticide Laboratory, Phytotoxicity Research Department, Agricultural Research Center, 7 Nadi El-Seid Street, Dokki, Giza 12618, Egypt

**Keywords:** *Urophorus humeralis*, morphometrics, larval instar, Dyar’s rule, integrated pest management

## Abstract

Effective integrated pest management (IPM) relies on precise knowledge of pest developmental biology, particularly the identification of larval instars, which is fundamental for predicting population dynamics and timing control interventions. This study established a morphometric framework for the larval staging of a sap beetle pest infesting pear orchards. Specimens were collected and reared under laboratory conditions, with their identity confirmed as *Urophorus humeralis* through integrated morphological and molecular (*COI* barcoding) analysis. To determine the number of larval instars, head capsule width (HCW), inter-antennal distance (IAD), and inter-caudal distance (ICD) were measured. Frequency distribution analysis and validation using Dyar’s rule via linear regression revealed three distinct larval instars. Head capsule width was identified as the most reliable and consistent morphological character for instar discrimination. This study reports for the first time the infestation of pear fruits by *U. humeralis* and provides detailed morphometric criteria for larval staging, delivering essential baseline data for the biology of Nitidulidae and a scientific basis for developing stage-specific pest management strategies.

## Simple Summary

This study provides the first report of *Urophorus humeralis* infesting pear fruits and establishes detailed morphometric criteria for the larval instars of this sap beetle. By measuring the larval three indicators—head capsule width (HCW), inter-antennal distance (IAD), and inter-caudal distance (ICD)—head capsule width was confirmed as the most reliable and stable morphological characteristic for distinguishing larval instars. Frequency distribution analysis and verification of Dyar’s rule (describes the phenomenon where the width of the clypeus in insects of adjacent age classes exhibits a geometric relationship) based on linear regression confirmed the existence of three distinct larval instars. It not only offers a valuable reference framework for the developmental classification of *U*. *humeralis*, but also enhances our understanding of its biological characteristics and life history (a comprehensive description of the series of developmental stages an individual goes through from birth to death), thereby supporting the refinement of pest monitoring systems and optimization of management measures at each developmental stage. The findings contribute to the development of more efficient and sustainable control strategies for sap-feeding beetles in pear orchard production systems.

## 1. Introduction

Sap beetles (Coleoptera: Nitidulidae) constitute a widely distributed and diverse family of insects, encompassing numerous species of considerable ecological and economic importance [1,2,3]. Their small body size, high adaptability, and overlapping generations contribute to their successful colonization of a wide range of habitats. Sap beetles exhibit diverse feeding habits; although many species are primarily fungivorous or frugivorous, their feeding activity can result in substantial crop damage and facilitate the transmission of plant pathogens, thereby classifying them as important agricultural and forestry pests [4,5,6,7,8,9]. In addition, several species visit flowers and act as pollinators, whereas others infest stored products such as dried fruits, traditional medicines, rice, and flour, making them significant stored-product pests [10,11,12,13,14]. Some species also occupy specialized ecological niches, including inhabiting the nests of other insects. A notable example is the small hive beetle, *Aethina tumida* Murray, a globally important quarantine pest whose larvae and adults parasitize honeybee colonies, feeding on honey and brood and causing severe economic losses; in extreme cases, infestations can lead to complete colony collapse [15].

In pest species, the larval stage is often the most destructive phase. Early instars generally possess softer cuticles and exhibit lower tolerance to pesticides, making them particularly vulnerable targets for control interventions [16,17,18]. Consequently, accurate identification of larval instars is essential for optimizing the timing of control measures and for understanding population dynamics. However, research on Nitidulidae has traditionally focused on adult taxonomy and phylogeny, whereas larval biology and instar determination remain comparatively understudied. For example, 2022, Han et al. divided the larval instars of *A. tumida*, indicating three instars. In the same year, Zhang Mingming et al. declared that *A. tumida* has four larval instars [19]. Dasgupta and Pal (2021) reported four larval instars in *Epuraea ocularis* [20], while Williams et al. (2021) provided the first description of the mature larva of *Omosita nearctica* without resolving its instar structure [21]. Earlier, Ortloff (2014) described the larval morphology of *Nitidula carnaria* based on the work of Hayashi et al. [22]; nevertheless, comprehensive morphometric approaches for larval instar discrimination in sap beetles are still scarce.

Currently, larval instar determination relies primarily on frequency distribution analysis of head capsule width (HCW), often validated using Dyar’s rule [23,24,25,26]. This empirical rule describes a geometric progression in the size of sclerotized structures, such as the head capsule, across successive molts. The conformity of observed measurements to this rule is commonly evaluated using Crosby’s growth constant, with values below 0.10 indicating a stable growth ratio and supporting the proposed instar classification [27]. Although HCW is widely accepted as a reliable diagnostic character, reliance on a single metric may limit resolution. Therefore, incorporating additional morphometric traits may improve the robustness and accuracy of instar discrimination.

In this context, the present study investigates a sap beetle species infesting pear orchards. The objectives are to: (1) accurately identify the species using an integrative approach combining morphological and molecular analyses, and (2) determine its larval instar structure by rearing larvae under laboratory conditions and applying morphometric analysis of head capsule width, inter-antennal distance, and caudal distance. By elucidating larval growth patterns and establishing reliable diagnostic criteria for instar classification, this study provides essential baseline data for the developmental biology and taxonomy of Nitidulidae and contributes to the development of stage-specific pest management strategies.

## 2. Materials and Methods

### 2.1. Insect Collection and Rearing Conditions

Adult *Urophorus humeralis* were collected from pear orchards in Wuyang County, Luohe City, Henan Province, China. Collected individuals were maintained under controlled laboratory conditions in an artificial climate chamber (HQH-H300, Shanghai Yuejin Medical Devices Co., Ltd., Shanghai, China) at “25 ± 1 °C, 75 ± 5%” relative humidity, and a 16:8 h light–dark photoperiod.

Adults were housed in plastic rearing boxes (17 cm × 11.5 cm × 4.7 cm) at a 1:1 male-to-female ratio. Fresh pear fruits were provided as both food and oviposition substrates and were replaced regularly. Following egg hatching, larvae were transferred to new rearing boxes and maintained under the same environmental conditions until experimental use.

### 2.2. Adult Morphological Identification

Adults were identified based on external morphological traits, including pronotum shape, elytral pattern, pronotal spine arrangement, and external genitalia of both sexes. Identification was conducted using standard taxonomic keys and reference works, including the *Catalogue of Palaearctic Coleoptera* and the *Beijing Beetle Ecological Atlas* [28,29,30].

### 2.3. Molecular Identification

#### 2.3.1. DNA Extraction and PCR Amplification

For molecular identification, individual adult specimens were placed in 1.5 mL microcentrifuge tubes, frozen in liquid nitrogen, and thoroughly homogenized. This procedure was performed for three biological replicates. Genomic DNA was extracted using the Universal Genomic DNA Extraction Kit (CWBIO, Shanghai, China) according to the manufacturer’s instructions.

A fragment of the mitochondrial cytochrome c oxidase subunit I (*COI*) gene was amplified using the primers *COI*-F (5′-CAACATTTATTTTGATTTTTTGG-3′) and *COI*-R (5′-GCACTAWTCTGCCATATTAGA-3′). PCR reactions were carried out in a 50 μL volume containing 4 μL DNA template, 25 μL 2× PCR Master Mix, 2 μL of each primer (10 μM), and 17 μL sterile dd H_2_O.

Thermal cycling was performed as follows: initial denaturation at 94 °C for 5 min; 35 cycles of denaturation at 94 °C for 30 s, annealing at 47 °C for 30 s, and extension at 72 °C for 2 min; followed by a final extension at 72 °C for 10 min. PCR products were purified and sequenced bidirectionally by Shanghai Sangon Biotech Co., Ltd., Shanghai, China.

#### 2.3.2. Sequence Analysis and Phylogenetic Reconstruction

Raw sequence data were assembled and edited using SeqMan Pro v11.1.0 [31]. Consensus sequences were queried against the NCBI database using BLASTn for species identification, and reference *COI* sequences of closely related taxa were retrieved from GenBank. Multiple sequence alignment was performed in MEGA v12 [32].

For phylogenetic reconstruction, the subfamily Carpophilinae was designated as the ingroup, with Epuraeinae and Promepotinae serving as outgroups. Phylogenetic analyses were conducted using Bayesian Inference (BI) in MrBayes v3.2 and Maximum Likelihood (ML) in IQ-TREE v2.0.4 [33,34,35]. BI analysis employed the GTR + F + G4 model, with two independent runs continued until the average standard deviation of split frequencies reached 0.004. ML analysis was performed using the TIM2 + F + R2 model with 5000 ultrafast bootstrap replicates. Final phylogenetic trees were visualized and annotated using iTOL v7 [36].

### 2.4. Larval Instar Determination

**Figure 1:**
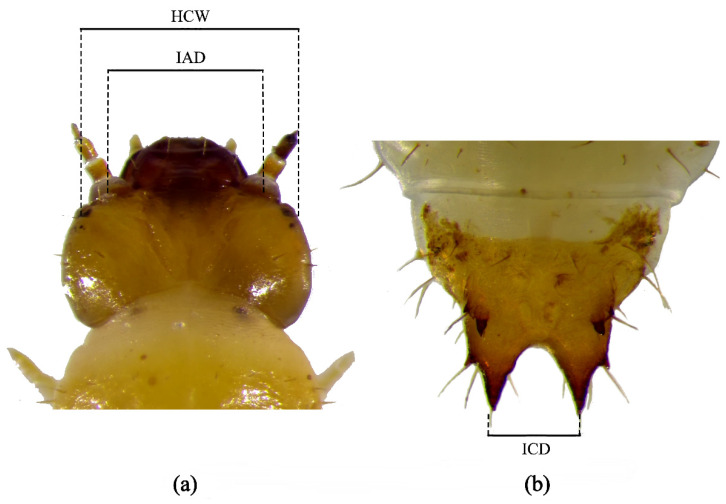
Morphometric illustration of *Urophorus humeralis* larva. (**a**) Head showing measurements of head capsule width (HCW) and inter-antennal distance (IAD). (**b**) Caudal process illustrating inter-caudal distance (ICD) used for instar determination.

Larval instars were determined using morphometric measurements of sclerotized body structures. Three morphological parameters were recorded: head capsule width (HCW), inter-antennal distance (IAD), and inter-caudal distance (ICD) (Figure 1; Table 1) [37,38]. The larval sampling amount covered all instars. Observations commenced immediately after egg hatching. Each day, 15 larvae were randomly selected and measured using a digital microscope (MOTIC K Series SZ51) equipped with a 20× objective and calibrated eyepiece scale. Measurements were collected daily until pupation.

#### Statistical Analysis

Frequency distribution analyses of head capsule width, inter-antennal distance, and inter-caudal distance were conducted using SPSS v26.0. The validity of larval instar classification was assessed following Crosby’s growth rule in combination with linear regression analysis [25,26]. According to Crosby’s growth rule, the Brooks index and Crosby index were calculated using the following equations [39]:

Crosby index = (*b_n_* − *b_n−_*_1_)/*b_n_*_−1_

Brooks index = *X_n_*_−1_/*X_n_*_−1_

where *b_n_* and *b_n_*_−1_ represent the Brooks indices of the nth and (n − 1)^th^ instars, respectively, and *X_n_* and *X_n_*_−1_ denote the mean morphometric values of the nth and (n − 1)^th^ instars.

A Crosby index < 0.10 was considered indicative of a valid separation between instars, whereas values > 0.10 suggested potential overlap. Final instar determination was based on the most reliable morphometric indicator, further corroborated by direct observations of molting events under laboratory conditions [40,41,42,43]. Larval morphology, including the head, abdomen, and caudal appendages, was documented using the MOTIC digital imaging system.

## 3. Results

### 3.1. Adult Characteristics

#### 3.1.1. Adult Morphological Characteristics

**Figure 2:**
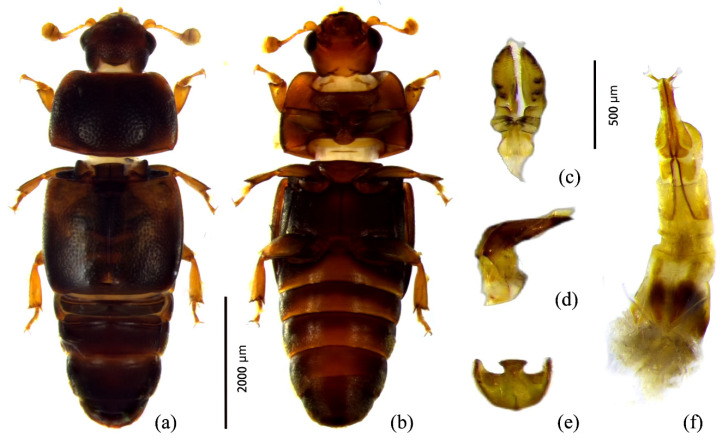
Adult morphology and external genitalia of *Urophorus humeralis*. (**a**) Dorsal view of male adult; (**b**) ventral view of male adult; (**c**) dorsal view of the tegmen; (**d**) lateral view of the tegmen; (**e**) eighth sternite and strut; (**f**) ventral view of the ovipositor.

Adults of *Urophorus humeralis* measured 4.2–4.5 mm in length and 1.4–1.5 mm in width, with an elongated-ovate body shape and a slightly convex dorsal surface. The body coloration ranged from dark brown to black; the head and mouthparts were reddish brown, the antennae reddish brown with a brownish antennal club, and the elytra bore indistinct yellowish-brown markings at the humeral region. The abdomen was reddish brown to brown, and the legs were reddish brown (Figure 2a).

The head exhibited fine, dense punctation with a smooth surface covered by fine yellow setae and was wider than long. Compound eyes were well developed. The antennal scape was constricted, and the antennae were approximately equal in length to head width. Each antenna consisted of 11 segments, with the eighth segment distinctly transverse and segments IX–XI forming a compact club; segments IX and X were similar in width, while the terminal segment had symmetrical apices.

The pronotum was nearly quadrate, with a slightly concave anterior margin and a nearly straight posterior margin. Anterior angles were bluntly rounded, posterior angles obtuse, and lateral margins convex with a slight median elevation. The surface was smooth, punctate, and sparsely covered with fine yellow setae. The scutellum was nearly pentagonal. The elytra were as wide at the base as the pronotum, with slightly protruding humeral angles. The apical margin was slightly inclined, the outer apical angle rounded, and the sutural angle slightly obtuse. Elytral punctures were large and sparse, bearing fine yellow setae.

The prosternum was keel-shaped, with a rough, densely punctate surface. The prosternal process was anteriorly protruding, widening toward the apex, which was rounded and lacked a terminal wall. The metasternum had a convex disc with minute punctures and lacked axillary plates.

**Figure 3:**
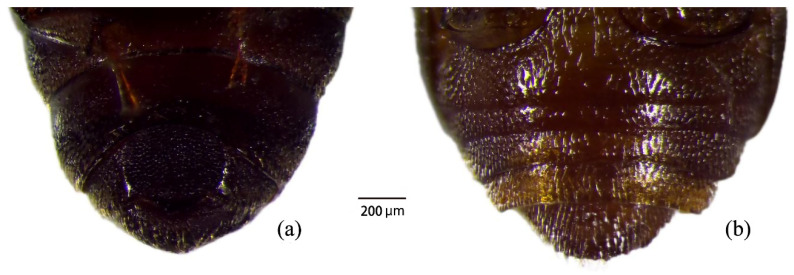
Indentation of the fifth abdominal sternite in *Urophorus humeralis*: (**a**) male; (**b**) female.

Females were generally larger than males. In males, the fifth abdominal sternite exhibited a nearly circular depression densely covered with punctures (Figure 3). Male genitalia showed strong sclerotization of the aedeagal base and the eighth abdominal sternite, particularly along the margins (Figure 2e). The median lobe terminated in a relatively flat apex. The aedeagal base was slender, bearing a distinct anterior tubercle and well-developed lateral setae (Figure 2c,d). Female genitalia consisted of a genital base plate with smooth lateral margins near the apex, fused distally and bearing a genital spine (Figure 2f).

#### 3.1.2. Adult Molecular Identification

Evolutionary distance analysis based on *COI* gene sequences (Table 2) showed that the intraspecific genetic distance between the tested specimens (*Urophorus* sp.) and *U. humeralis* was 0.00, markedly lower than the minimum interspecific threshold (0.20) for species delimitation. The average intraspecific genetic distance was 0.00, whereas interspecific distances ranged from 0.14 to 0.23, with a mean of 0.17. Genetic distances within species of Nitidulidae were consistently lower than those between species, indicating that the *COI* gene provides reliable resolution for sap beetle identification.

**Figure 4:**
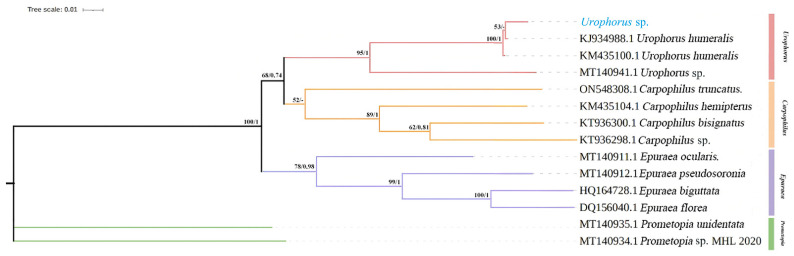
Maximum Likelihood (ML) and Bayesian Inference (BI) phylogenetic trees based on *COI* gene sequences. Numbers at the nodes indicate bootstrap values (BV) for ML and posterior probabilities (PP) for BI analyses.

Both Maximum Likelihood (ML) and Bayesian Inference (BI) analyses produced identical phylogenetic topologies (Figure 4). The subfamily Carpophilinae was clearly separated from the outgroups Epuraeinae and Promepotinae (posterior probability, PP = 1; bootstrap value, BV = 100). The two genera within Carpophilinae formed a monophyletic clade with moderate support (PP = 0.74; BV = 65). The tested specimens and reference sequences of *U. humeralis* clustered into a single, well-supported clade (PP = 1; BV = 100), confirming their species identity.

### 3.2. Classification of Larval Instars

**Figure 5:**
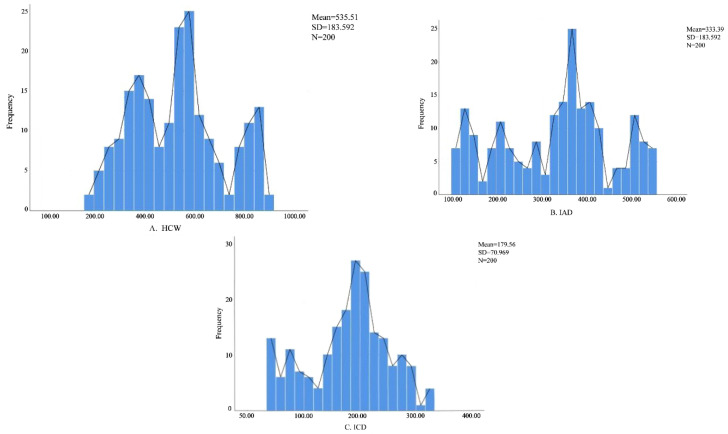
Frequency distribution of morphometric measurements of *Urophorus humeralis* larvae: (**A**) head capsule width (HCW), (**B**) inter-antennal distance (IAD), and (**C**) inter-caudal distance (ICD).

A total of 200 measurements were obtained for head capsule width (HCW), inter-antennal distance (IAD), and inter-caudal distance (ICD). Frequency distribution analysis of HCW revealed three distinct peaks (Figure 5), suggesting the presence of three larval instars. In contrast, IAD and ICD did not show clear multimodal distributions and were therefore considered less suitable for instar discrimination.

#### 3.2.1. Crosby Index Analysis

Morphometric statistics for each larval instar, including sample size, coefficient of variation, Brooks index, and Crosby index, are presented in Table 3. The mean HCW values for first-, second-, and third-instar larvae were 395.28 µm, 590.94 µm, and 821.07 µm, respectively. Significant differences were observed among instars for all morphological indicators. Crosby index values were consistently below 0.10, indicating that classification into three larval instars was statistically valid.

#### 3.2.2. Relationship Between Morphometric Indicators and Instar Stage

Regression analysis revealed highly significant relationships between instar stage and mean HCW values (Table 4). Among the tested models, cubic and quadratic regressions produced the highest coefficients of determination (R^2^), followed by the linear model, while the exponential model showed the weakest fit. These results confirm that HCW is the most reliable morphometric indicator for larval instar determination, consistent with the frequency distribution and Crosby index analyses.

#### 3.2.3. Larval Morphological Characteristics

**Figure 6:**
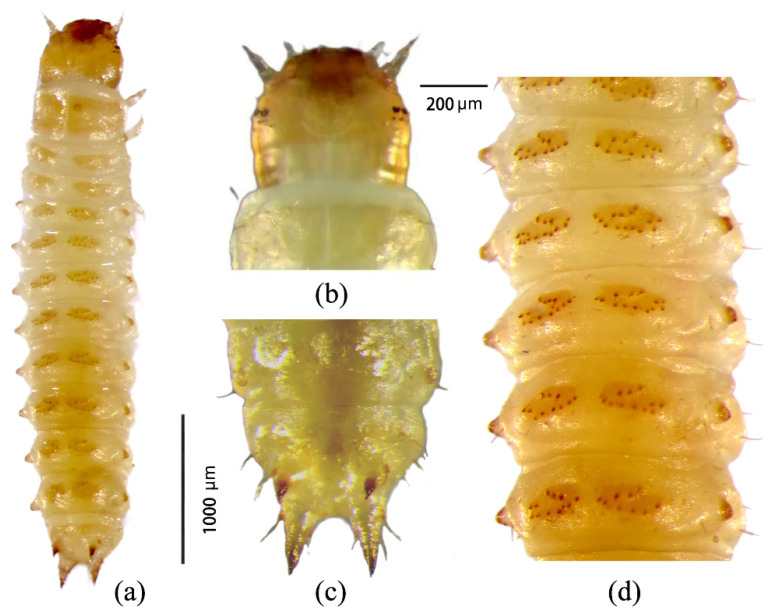
Morphological features of first-instar larvae of *Urophorus humeralis*: (**a**) dorsal surface, (**b**) head capsule, (**c**) ninth abdominal segment showing buccal and caudal processes, and (**d**) abdominal segments with dorsal ossicles.

***First instar.*** Body length ranged from 3.4 to 4.2 mm, with a developmental duration of 1–2 days. Larvae were milky white and translucent, with a spindle-shaped body gradually tapering posteriorly (Figure 6a). The head capsule showed low sclerotization and a glossy, slightly wrinkled surface. The frontal suture was incompletely fused, forming a distinct U-shaped structure (Figure 6b). The abdomen consisted of 10 segments, with a marked constriction at the ninth segment, where the buccal and caudal processes were located (Figure 6c). The buccal process was shorter and less sclerotized than the caudal process. Each abdominal segment bore a pair of dorsal elliptical ossicles, each with 12–13 spines (Figure 6d).

**Figure 7:**
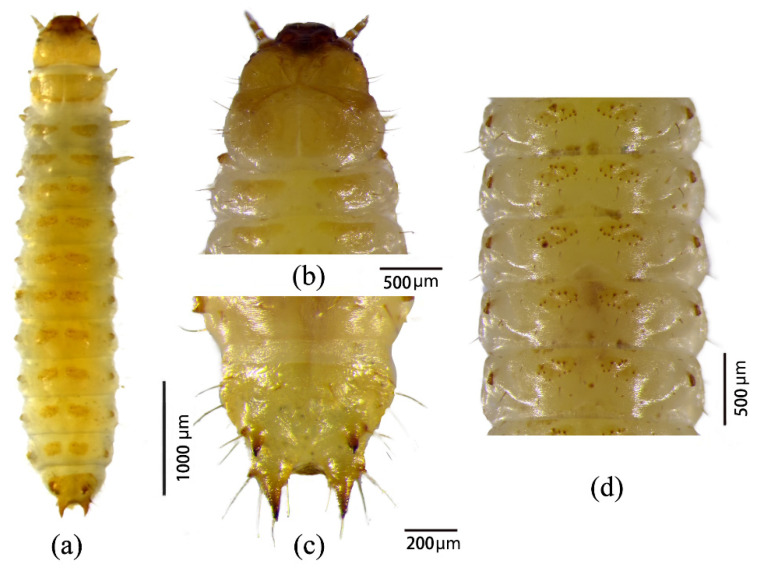
Morphology of second-instar larvae of *Urophorus humeralis*: (**a**) dorsal view, (**b**) head capsule, (**c**) ninth abdominal segment with buccal and caudal processes, and (**d**) abdominal segments showing dorsal ossicles.

***Second instar.*** Body length ranged from 5.7 to 6.3 mm, with a developmental duration of 3–4 days. Larvae were pale yellowish white and larger than first-instar individuals (Figure 7a). The head capsule exhibited well-developed furrows and increased sclerotization, with advanced fusion of the frontal suture (Figure 7b). Both buccal and caudal processes at the ninth abdominal segment were strongly sclerotized (Figure 7c). Each dorsal abdominal ossicle bore 11–12 spines (Figure 7d), slightly fewer than in the first instar.

**Figure 8:**
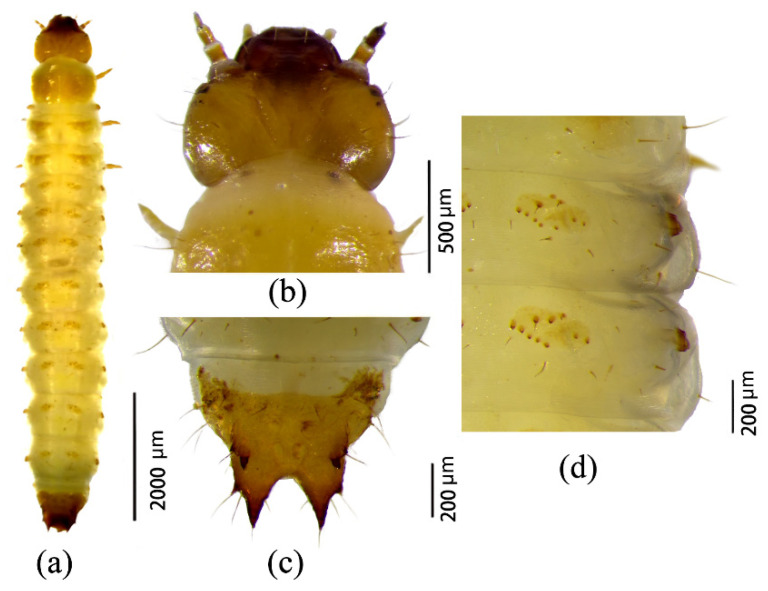
Morphology of third-instar larvae of *Urophorus humeralis*: (**a**) dorsal view, (**b**) head capsule, (**c**) ninth abdominal segment showing ossified buccal and caudal processes, and (**d**) abdominal segments with dorsal ossicles.

***Third instar.*** Body length ranged from 8.2 to 8.9 mm, with a developmental duration of 6–8 days. Larvae were yellowish white (Figure 8a). The head capsule displayed prominent ridges and high sclerotization, with the frontal suture nearly completely fused (Figure 8b). The ninth abdominal segment was almost entirely sclerotized, and both buccal and caudal processes were fully developed (Figure 8c). Each dorsal abdominal ossicle bore 9–10 spines (Figure 8d), indicating a further reduction compared to earlier instars.

### 3.3. Morphological Characteristics of Eggs and Pupae

**Figure 9:**
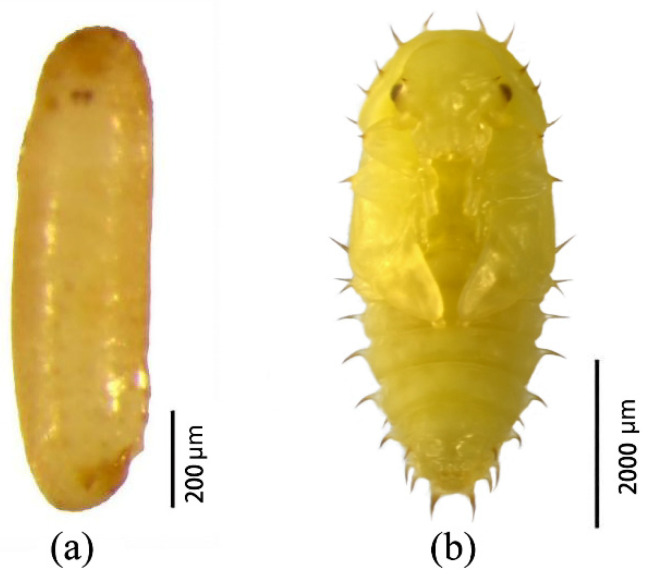
Morphology of *Urophorus humeralis* immature stages: (**a**) egg showing kidney shape and smooth to slightly rough surface, (**b**) exarate pupa with spines on head, thorax, and abdomen.

***Eggs.*** Eggs measured 1.0–1.1 mm in length and 0.2–0.3 mm in width and were laid singly. The incubation period lasted 3–5 days. Eggs were kidney-shaped, initially transparent, gradually becoming opaque with a smooth to slightly rough surface, and turning creamy yellow in later stages (Figure 9a).

***Pupae.*** Pupae were exarate, measuring 3.5–4.2 mm in length and 1.1–1.4 mm in width, with a pupal duration of 5–7 days. Initially milky white and glossy, pupae gradually became creamy yellow, with progressive darkening of the compound eyes. The head, thorax, and abdomen were covered with thick, smooth spines. The head bore a pair of robust spines above the compound eyes. Abdominal segments II–VI each carried two pairs of spines: a longer pair along the ventral midline and a shorter pair along the dorsal midline (Figure 9b).

## 4. Discussion

Accurate species identification is a fundamental prerequisite for both basic biological research and the development of effective pest management strategies. Traditionally, sap beetle identification has relied heavily on external morphological characters. However, the small body size, morphological conservatism, and high interspecific similarity within Nitidulidae often make reliable identification difficult, particularly for non-specialists. In this context, molecular tools provide a powerful complementary approach, offering higher resolution, objectivity, and reproducibility for species delimitation and phylogenetic inference [44,45,46,47]. Among molecular markers, the mitochondrial cytochrome c oxidase subunit I (*COI*) gene has been widely adopted as a standard DNA barcode for insects and has proven highly effective for species identification and evolutionary studies in Coleoptera [48,49,50].

In the present study, an integrative taxonomic framework combining classical morphology and *COI*-based molecular analysis was employed to identify the sap beetle specimens. Morphological examination of diagnostic characters, including external body structures and genitalia, was fully consistent with the original description of *Urophorus humeralis*. This morphological identification was strongly corroborated by molecular evidence, in the constructed genetic distance table, the intraspecific genetic distance between the tested specimen (*Urophorus* sp.) and *U. humeralis* was much smaller than the minimum interspecific genetic distance threshold (0.20), which revealed zero genetic divergence between the tested specimens and reference sequences of *U. humeralis*. Furthermore, both Bayesian and maximum likelihood phylogenetic analyses placed the specimens in a well-supported monophyletic clade with *U. humeralis*, clearly separated from other closely related taxa. The results of molecular data analysis fully support the conclusion that the sap beetle is *U. humeralis*. The complete congruence between morphological and molecular results provides unequivocal confirmation of species identity and highlights the utility of integrative approaches for resolving taxonomic ambiguities in morphologically conservative insect groups.

Beyond species identification, understanding the developmental biology of pest insects is essential for designing rational and stage-specific control strategies. Both the larval and adult stages are the key period for causing economic damage because they feed on the pear fruit. In August, we first discovered that *U. humeralis* had emerged and inflicted significant damage in a pear orchard in Henan Province, China, primarily affecting the pear fruits. The orchard was intercropped with peach and pear trees, leading to the speculation that the pest had harmed peaches in July before descending into the soil. As the pears ripened, the pest then moved to the pear trees to cause damage. We documented the complete life cycle of *U. humeralis* under laboratory conditions, comprising an egg stage of 3–5 days, a larval stage of 10–14 days, and a pupal stage of 5–7 days. So, mid-July to the end of July should be the main period for the mature larvae of *U. humeralis* to come out from the fruit and then pupate in the soil. And also, this is the key period for controlling them. Therefore, accurate determination of larval instars is critical for optimizing the timing of monitoring programs and control interventions.

Using Dyar’s rule and morphometric analyses, we clearly distinguished three larval instars in *U. humeralis*. Among the evaluated morphological parameters, the inter-antennal distance (IAD) and inter-caudal distance (ICD) showed an obvious multi-peak distribution which is different from the head capsule width (HCW) with three obvious peaks. So the head capsule width (HCW) emerged as the most reliable and informative indicator for instar classification. Frequency distribution analysis, along with Crosby and Brooks indices, confirmed that HCW exhibited distinct multimodal patterns and consistent growth ratios between instars. In contrast, inter-antennal distance (IAD) and inter-caudal distance (ICD) showed higher variability. This discrepancy is likely attributable to the relatively low degree of sclerotization in larval appendages and the potential for measurement error caused by body contraction and postural variability, which can obscure subtle size differences [51]. These findings are consistent with previous studies on other beetle species, in which HCW has been repeatedly validated as the most stable and reliable metric for larval instar determination [26,52].

In addition to quantitative morphometrics, qualitative morphological traits also provided valuable diagnostic information. The progressive increase in sclerotization of the head capsule and caudal processes, along with the gradual reduction in the number of spines on abdominal ossicles across instars, offers practical morphological markers for rapid field and laboratory identification of larval stages. The detailed descriptions of egg and pupal morphology presented in this study further complement existing taxonomic resources and provide, for the first time, a comprehensive morphological reference for all developmental stages of *U. humeralis*.

From an applied perspective, the biological and ecological characteristics of *U. humeralis* pose significant challenges for pest management. Both larvae and adults are endocarp feeders that develop within pear fruit tissues, rendering early infestations difficult to detect and limiting the effectiveness of conventional chemical control. Moreover, concerns regarding pesticide residues on fresh produce and the restricted penetration of insecticides into pear fruit tissues further constrain chemical intervention. Under these conditions, the success of management programs depends largely on early detection, accurate stage-specific identification, and precise timing of control measures.

Therefore, the integrative taxonomic and developmental framework established in this study has direct practical implications for pest monitoring and management. By enabling reliable species identification and precise larval instar classification, these findings provide a scientific basis for improving surveillance protocols, optimizing intervention timing, and ultimately developing more sustainable and targeted control strategies for *U. humeralis*. In a broader context, this work demonstrates the value of combining molecular taxonomy with detailed developmental biology to address both fundamental and applied challenges in agricultural entomology.

## 5. Conclusions

Molecular identification, owing to its high specificity and reliability, represents a powerful complement to traditional morphological taxonomy. In this study, the integration of *COI*-based molecular analysis with detailed morphological examination enabled the accurate identification of *Urophorus humeralis* as a key pest infesting pear orchards. Moreover, the comprehensive characterization of larval instars, based on robust morphometric criteria, provides a valuable reference framework for developmental classification in *U. humeralis* and potentially for closely related species. This information not only enhances our understanding of the species’ biology and life history but also establishes a methodological foundation for future ecological, physiological, and population studies. Collectively, these findings contribute essential baseline knowledge for improving pest monitoring, refining stage-specific management practices, and ultimately supporting the development of more effective and sustainable control strategies for sap beetle infestations in pear fruit production systems.

## Figures and Tables

**Table 1:** Morphometric measurements of larval instars of *Urophorus humeralis*.

Name	Acronym	Description
Head capsule width	HCW	The horizontal distance between the outer edges of the two compound eyes on the back of the head
Inter-antennal distance	IAD	The lateral distance between the two antennae segments
Inter-caudal distance	ICD	Lateral distance between the tips of the two tails

**Table 2:** Genetic distance among 14 sap beetle species based on *COI* gene sequences.

Species	1	2	3	4	5	6	7	8	9	10	11	12	13
1. *Urophorus* sp.													
2. KJ934988.1 *Urophorus humeralis*	0.00												
3. KM435100.1 *Urophorus humeralis*	0.00	0.00											
4. MT140941.1 *Urophorus* sp.	0.11	0.11	0.11										
5. ON548308.1 *Carpophilus truncatus*	0.14	0.14	0.14	0.17									
6. KM435104.1 *Carpophilus hemipterus*	0.15	0.15	0.15	0.16	0.14								
7. KT936300.1 *Carpophilus bisignatus*	0.15	0.15	0.15	0.15	0.16	0.11							
8. KT936298.1 *Carpophilus* sp.	0.15	0.15	0.15	0.16	0.14	0.12	0.09						
9. MT140911.1 *Epuraea ocularis*	0.15	0.15	0.15	0.16	0.17	0.15	0.16	0.15					
10. MT140912.1 *Epuraea pseudosoronia*	0.15	0.15	0.15	0.15	0.16	0.15	0.15	0.15	0.14				
11. DQ156040.1 *Epuraea florea*	0.16	0.16	0.16	0.17	0.17	0.17	0.16	0.17	0.15	0.11			
12. HQ164728.1 *Epuraea biguttata*	0.18	0.18	0.18	0.17	0.18	0.18	0.16	0.18	0.15	0.11	0.07		
13. MT140934.1 *Prometopia* sp. MHL-2020	0.21	0.21	0.21	0.23	0.21	0.20	0.21	0.20	0.20	0.22	0.23	0.23	
14. MT140935.1 *Prometopia unidentata*	0.23	0.23	0.23	0.23	0.22	0.23	0.21	0.23	0.20	0.22	0.24	0.25	0.20

**Table 3:** Morphometric measurements and statistical parameters used for larval instar determination in the sap beetle.

Variable	Instar	Head Capsule Width Average Value	Sample Number	Variation Coefficient	Brooks Index	Crosby Index
Each instar	1	395.28 μm	45	0.1506	-	-
	2	590.94 μm	84	0.0900	1.4950	-
	3	821.07 μm	71	0.0607	1.3894	−0.0706

**Table 4:** Regression analysis between larval instar number and morphometric variables in *Urophorus humeralis*.

Variable	Regression Model	Regression Equation	Correlation Coefficient (R^2^)	Significance
Head capsule width	Linear	y = 3.4316x + 147.83	R^2^ = 0.9668	*p* < 0.001
	Quadratic polynomial	y = −0.0025x^2^ + 3.9513x + 129.73	R^2^ = 0.9682	
	Cube polynomial	y = 0.0001x^3^ − 0.0419x^2^ + 7.2415x + 72.014	R^2^ = 0.9786	
	Index	y = 197.71e^0.008x^	R^2^ = 0.9167	

## Data Availability

The authors confirm that the data supporting the findings of this study are available in this paper and its Supplementary Materials. The original contribution sequences have been uploaded to the GenBank public repository under accession number: PX974060.

## Supplementary Materials

The following supporting information can be downloaded at: https://www.mdpi.com/article/10.3390/insects17030344/s1. Figure S1: Photographs of Urophorus humeralis adults and larvae at different instars. File S1: The experimental data of head capsule width, inter-antennal distance and inter-caudal distance were measured. Sequence S1: DNA sequence of Urophorus humeralis.
