# Supplementary figures and images for: Determination of the Morphometric Characteristics of Larval Instars in the Sap Beetle Urophorus humeralis (Coleoptera: Nitidulidae)
Source: Insects. 2026 Mar 21;17(3):344. doi: 10.3390/insects17030344 (PMC13026160; doi:10.3390/insects17030344)

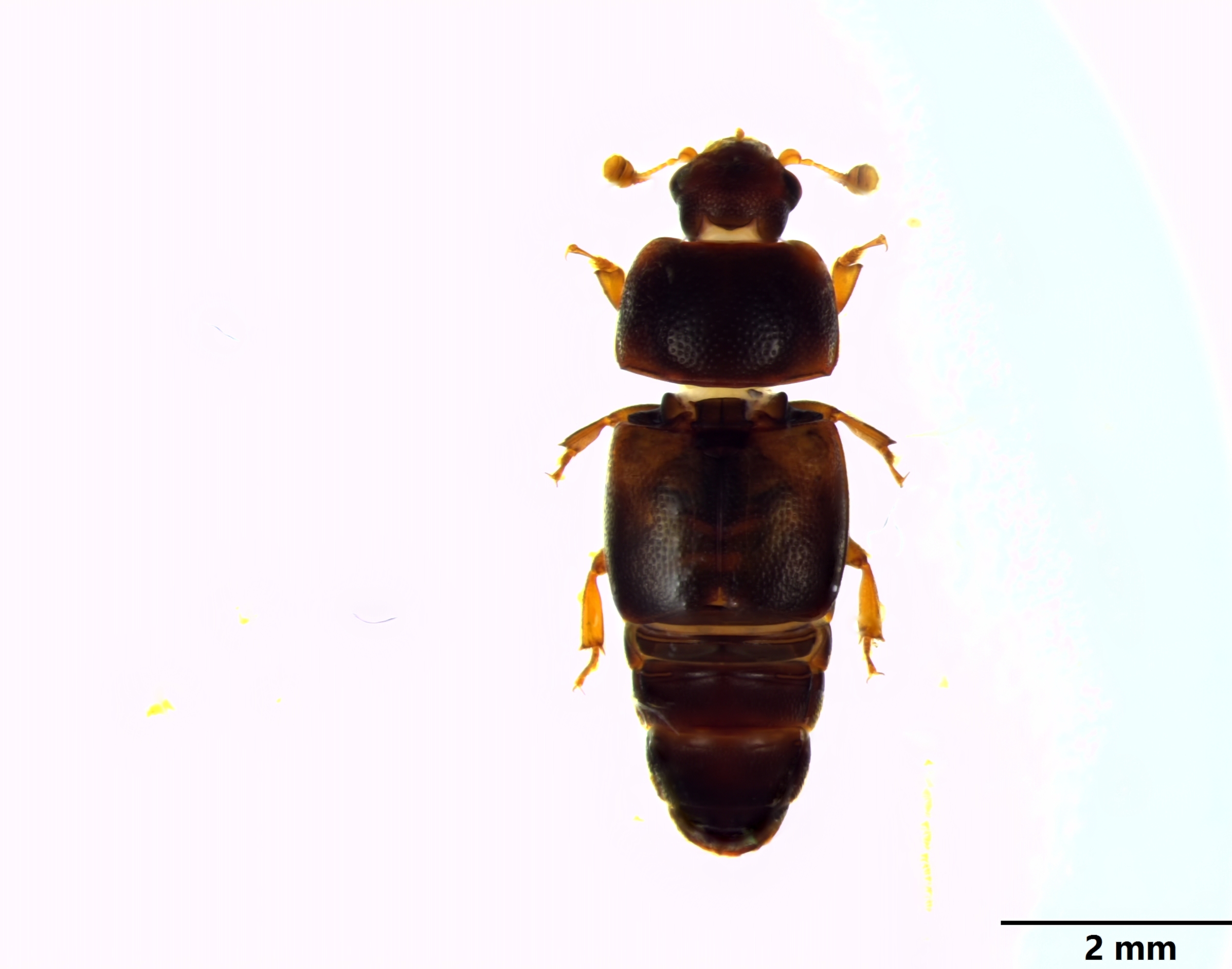

Supplement: Supplementary file 1 [file insects-17-00344-s001.zip › Experimental Data on Urophorus humeralis Nails/Figure/ Adult/Adult/Dorsal view of male adult.jpg]

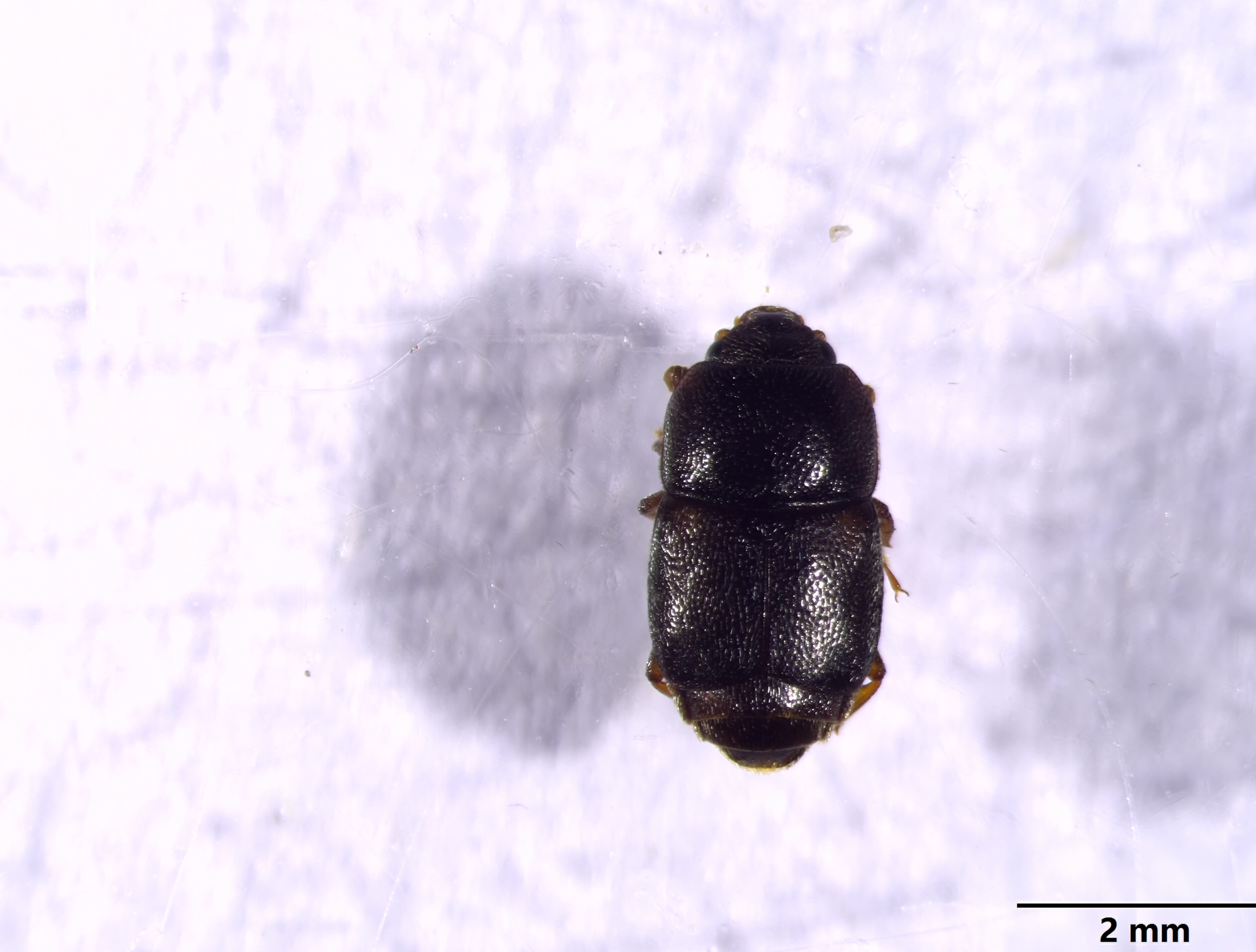

Supplement: Supplementary file 1 [file insects-17-00344-s001.zip › Experimental Data on Urophorus humeralis Nails/Figure/ Adult/Adult/female.tif]

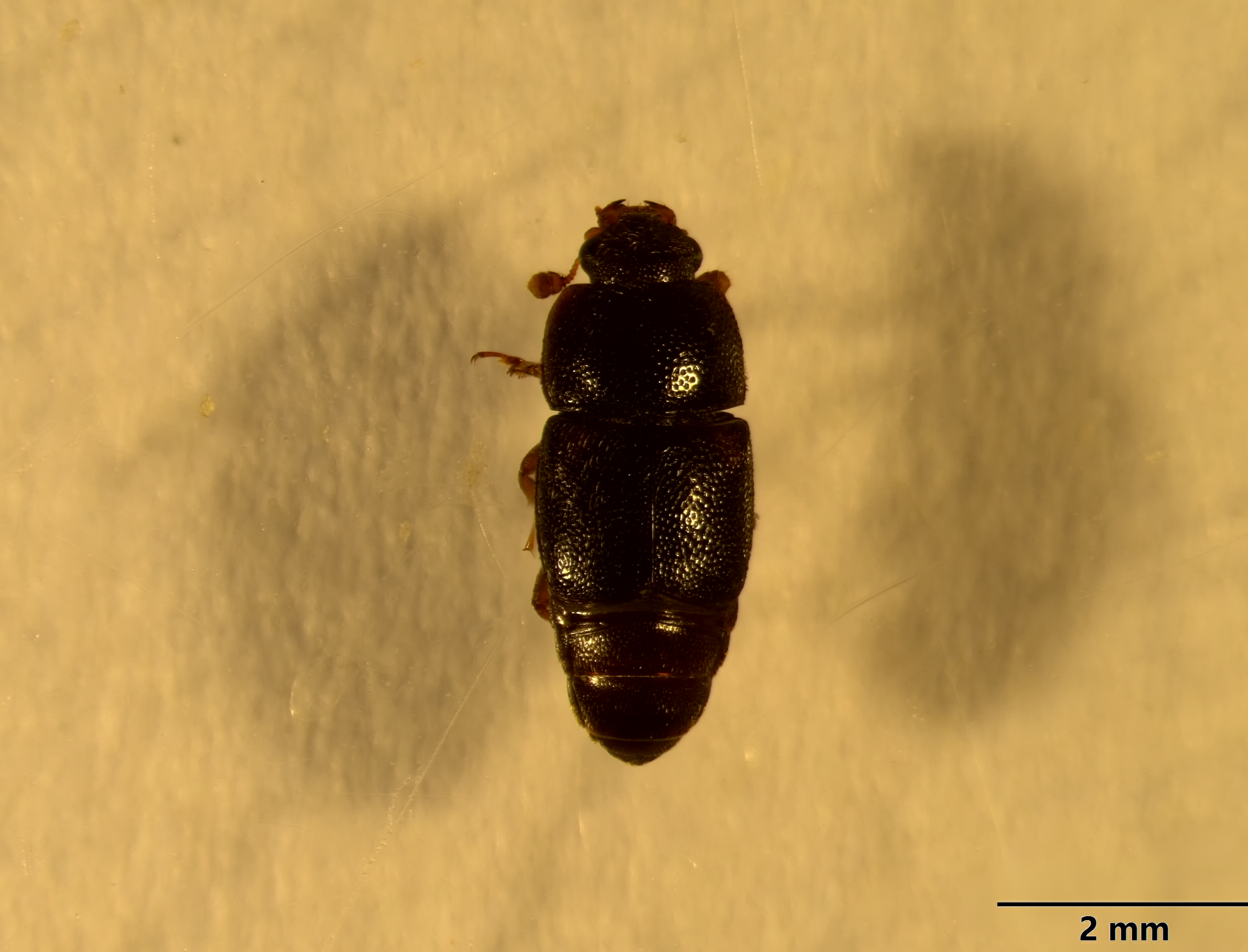

Supplement: Supplementary file 1 [file insects-17-00344-s001.zip › Experimental Data on Urophorus humeralis Nails/Figure/ Adult/Adult/male.tif]

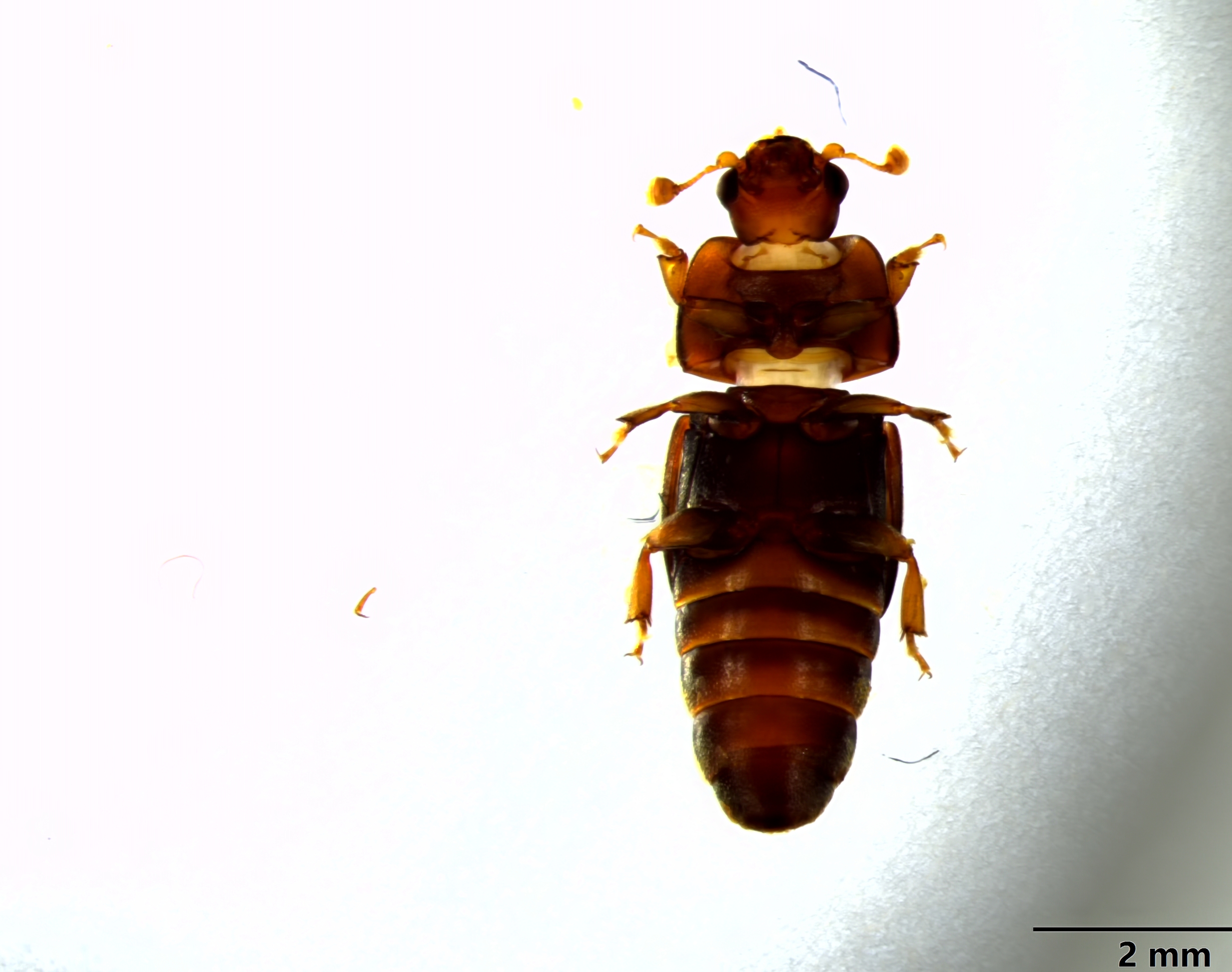

Supplement: Supplementary file 1 [file insects-17-00344-s001.zip › Experimental Data on Urophorus humeralis Nails/Figure/ Adult/Adult/ventral view of male adult.jpg]

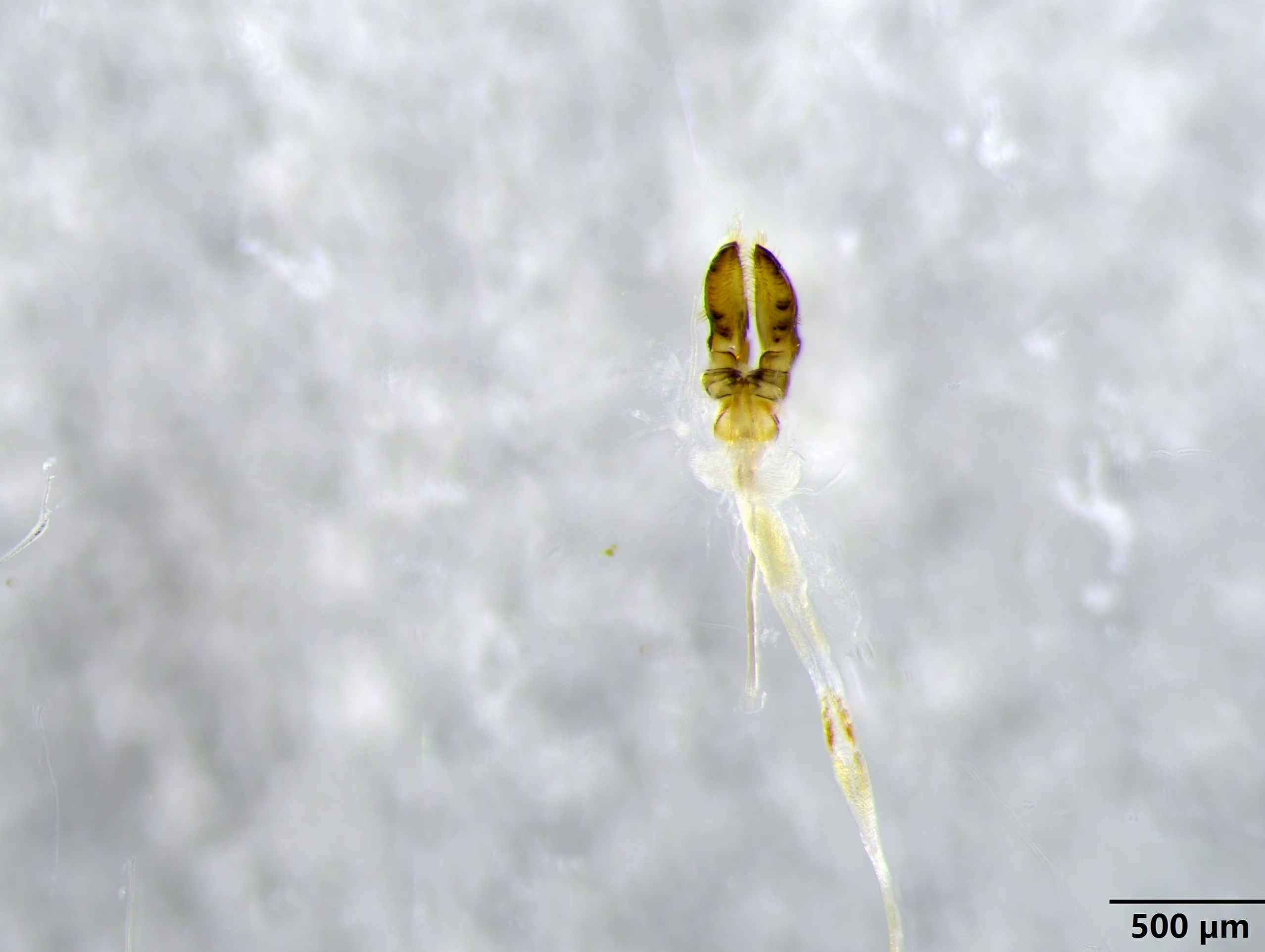

Supplement: Supplementary file 1 [file insects-17-00344-s001.zip › Experimental Data on Urophorus humeralis Nails/Figure/ Adult/external genitalia/dorsal view of the tegmen.jpg]

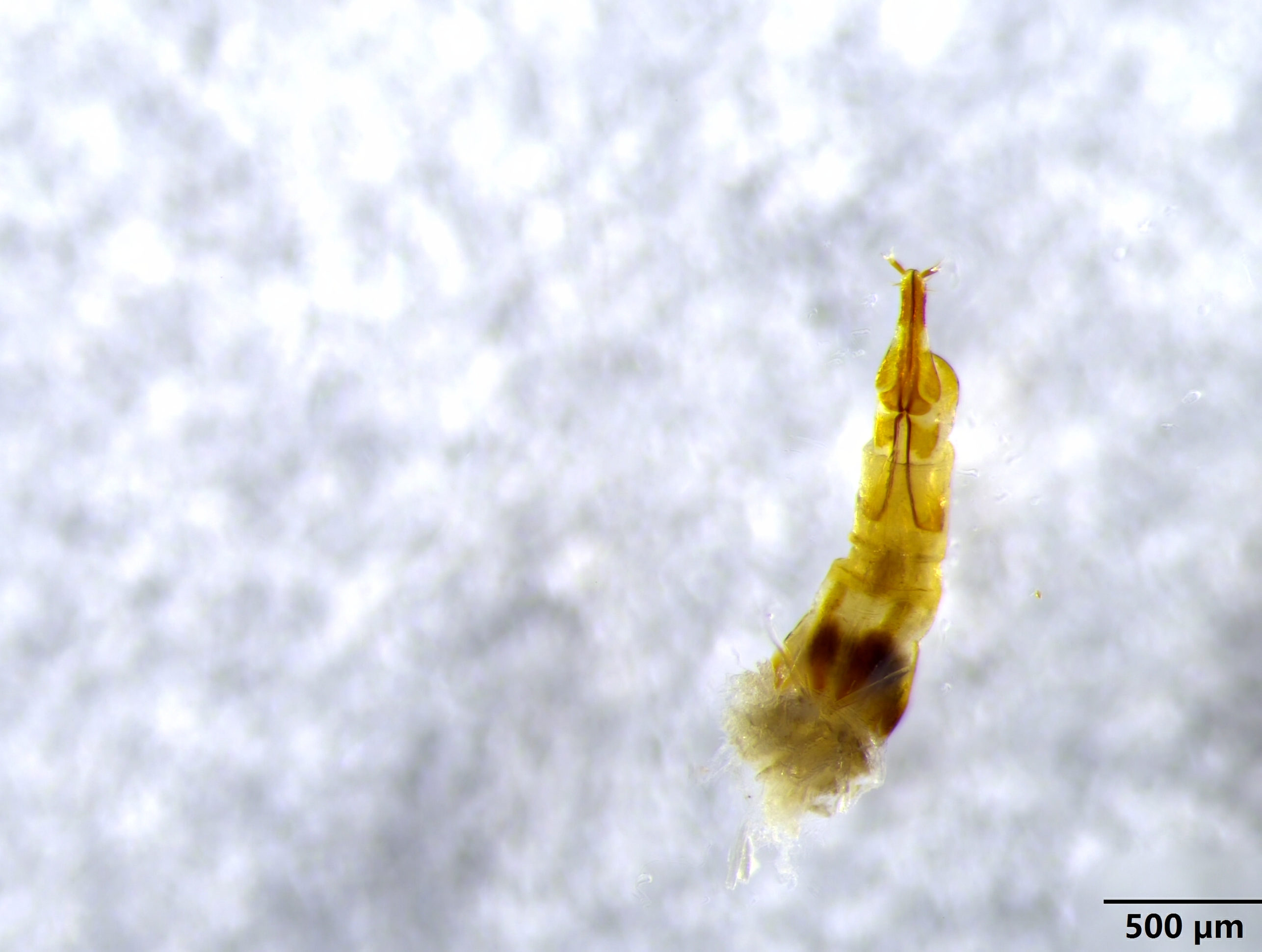

Supplement: Supplementary file 1 [file insects-17-00344-s001.zip › Experimental Data on Urophorus humeralis Nails/Figure/ Adult/external genitalia/ventral view of the ovipositor.jpg]

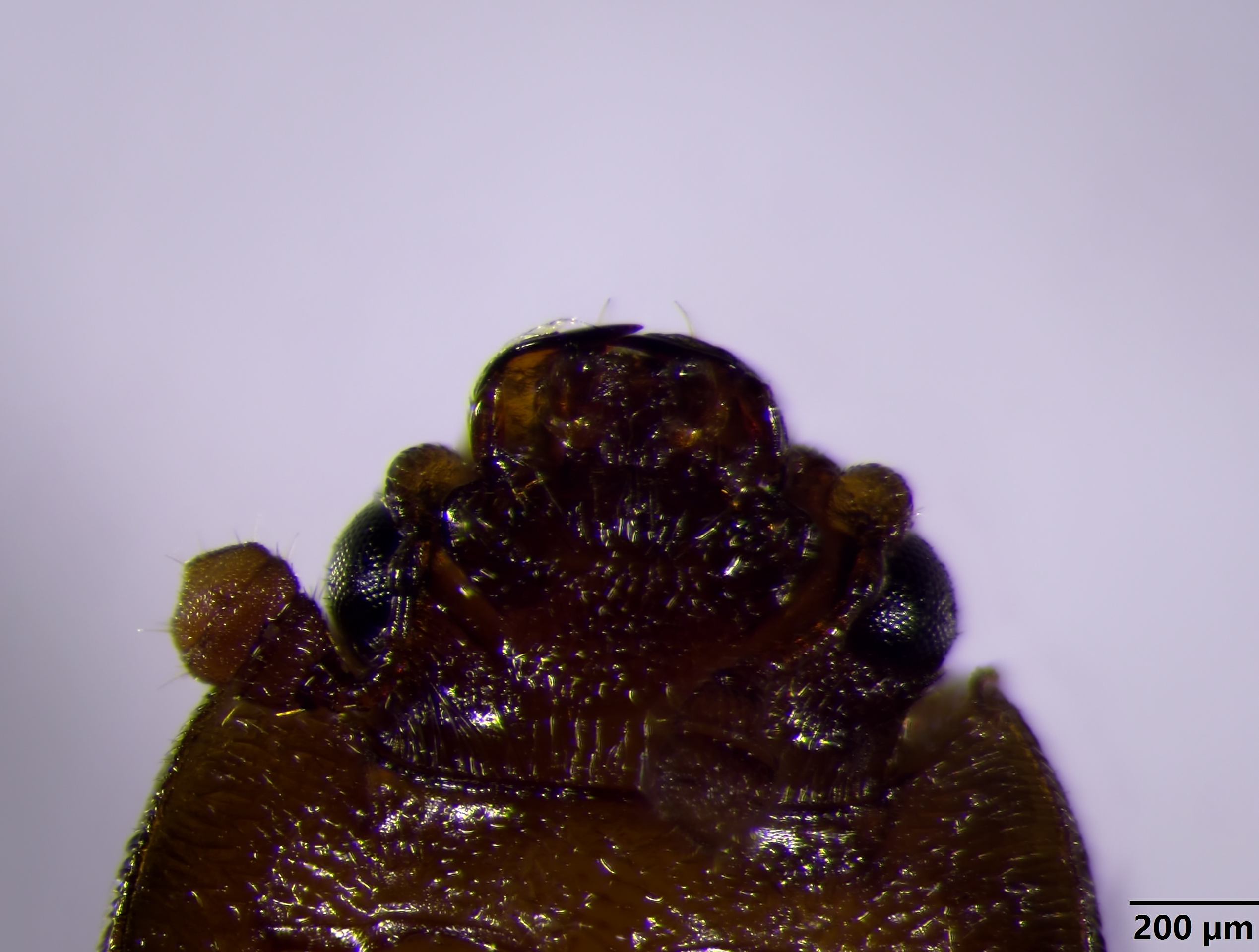

Supplement: Supplementary file 1 [file insects-17-00344-s001.zip › Experimental Data on Urophorus humeralis Nails/Figure/ Adult/head/Female head.tif]

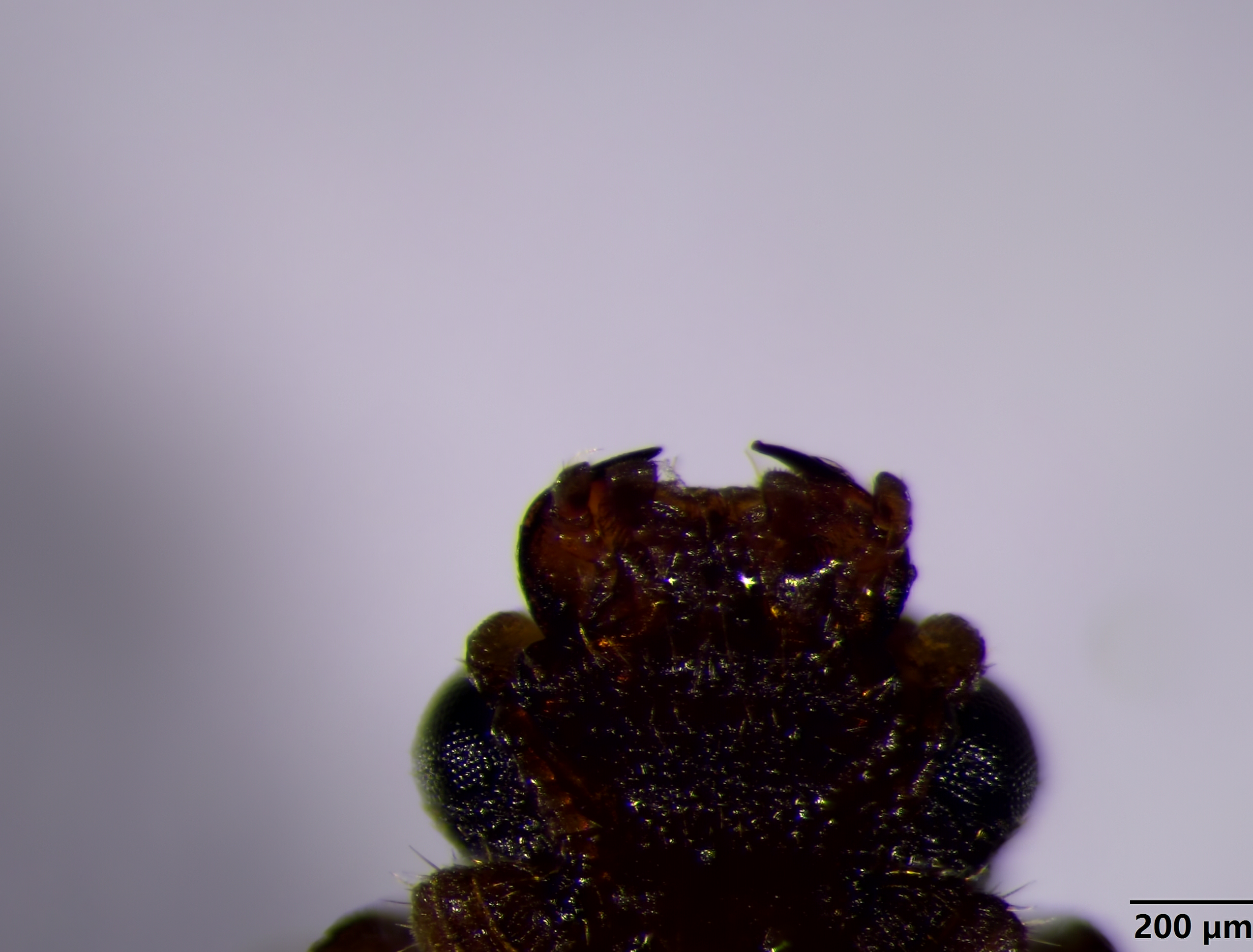

Supplement: Supplementary file 1 [file insects-17-00344-s001.zip › Experimental Data on Urophorus humeralis Nails/Figure/ Adult/head/Male head.tif]

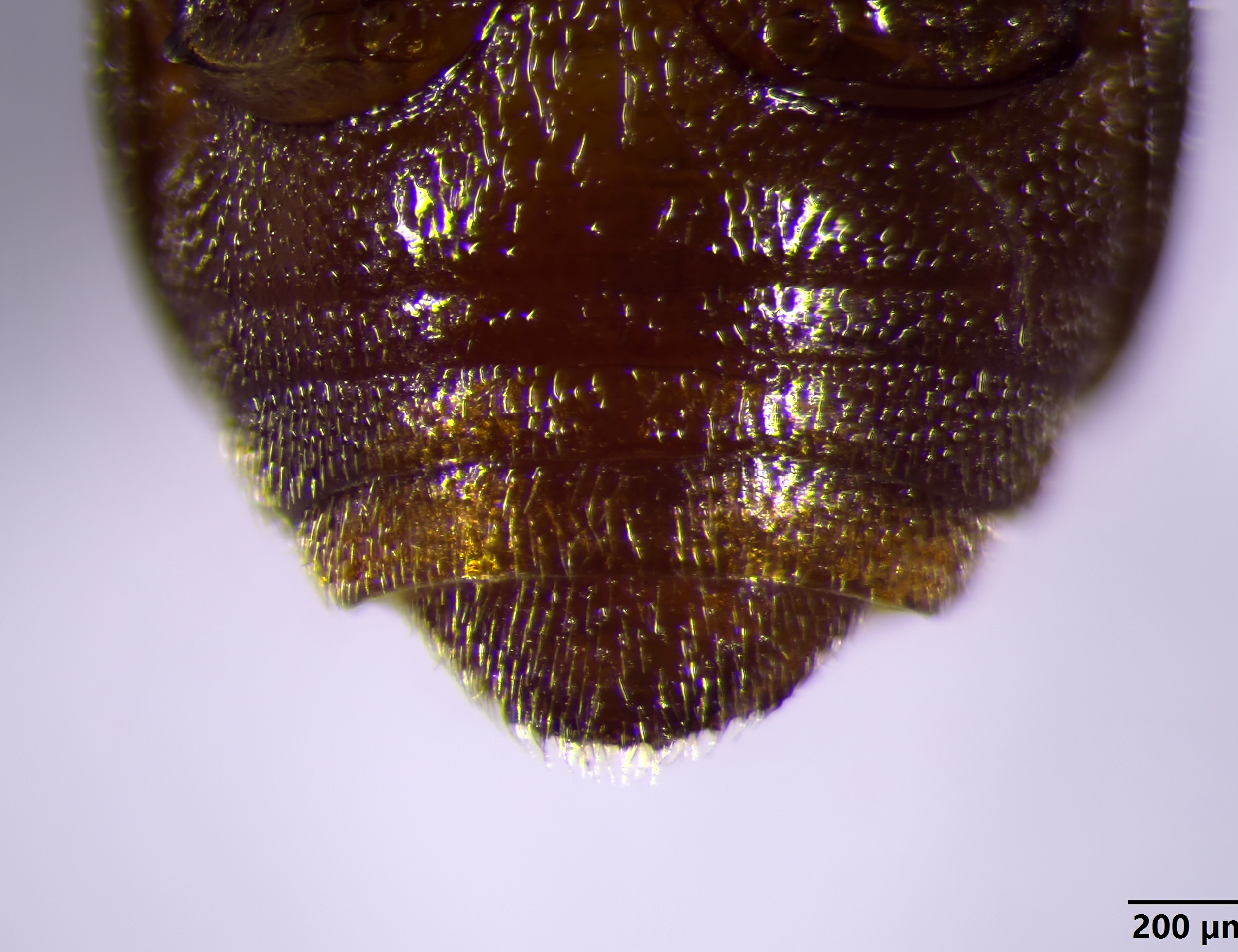

Supplement: Supplementary file 1 [file insects-17-00344-s001.zip › Experimental Data on Urophorus humeralis Nails/Figure/ Adult/Indentation of the fifth abdominal sternite / female.tif]

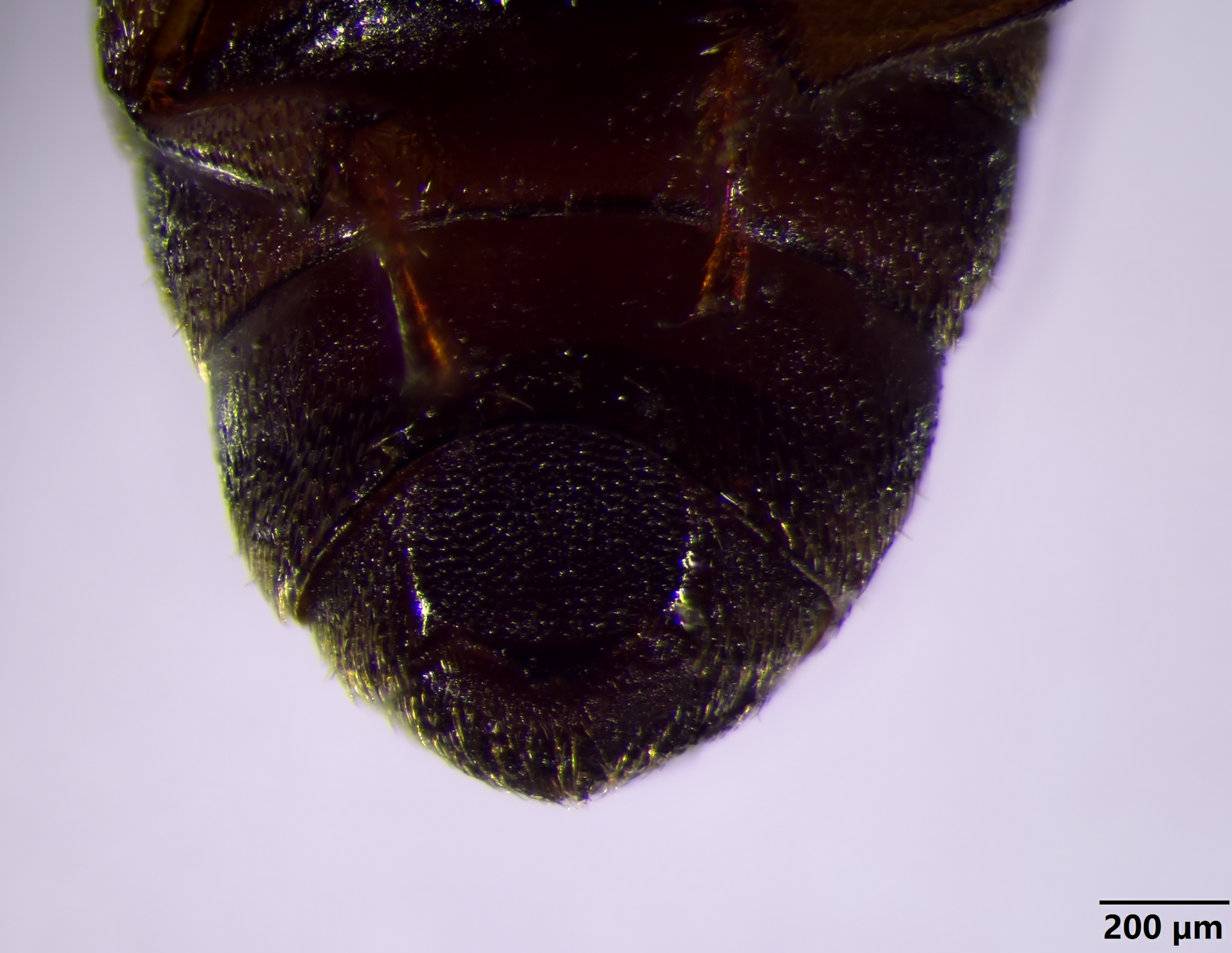

Supplement: Supplementary file 1 [file insects-17-00344-s001.zip › Experimental Data on Urophorus humeralis Nails/Figure/ Adult/Indentation of the fifth abdominal sternite /male.tif]

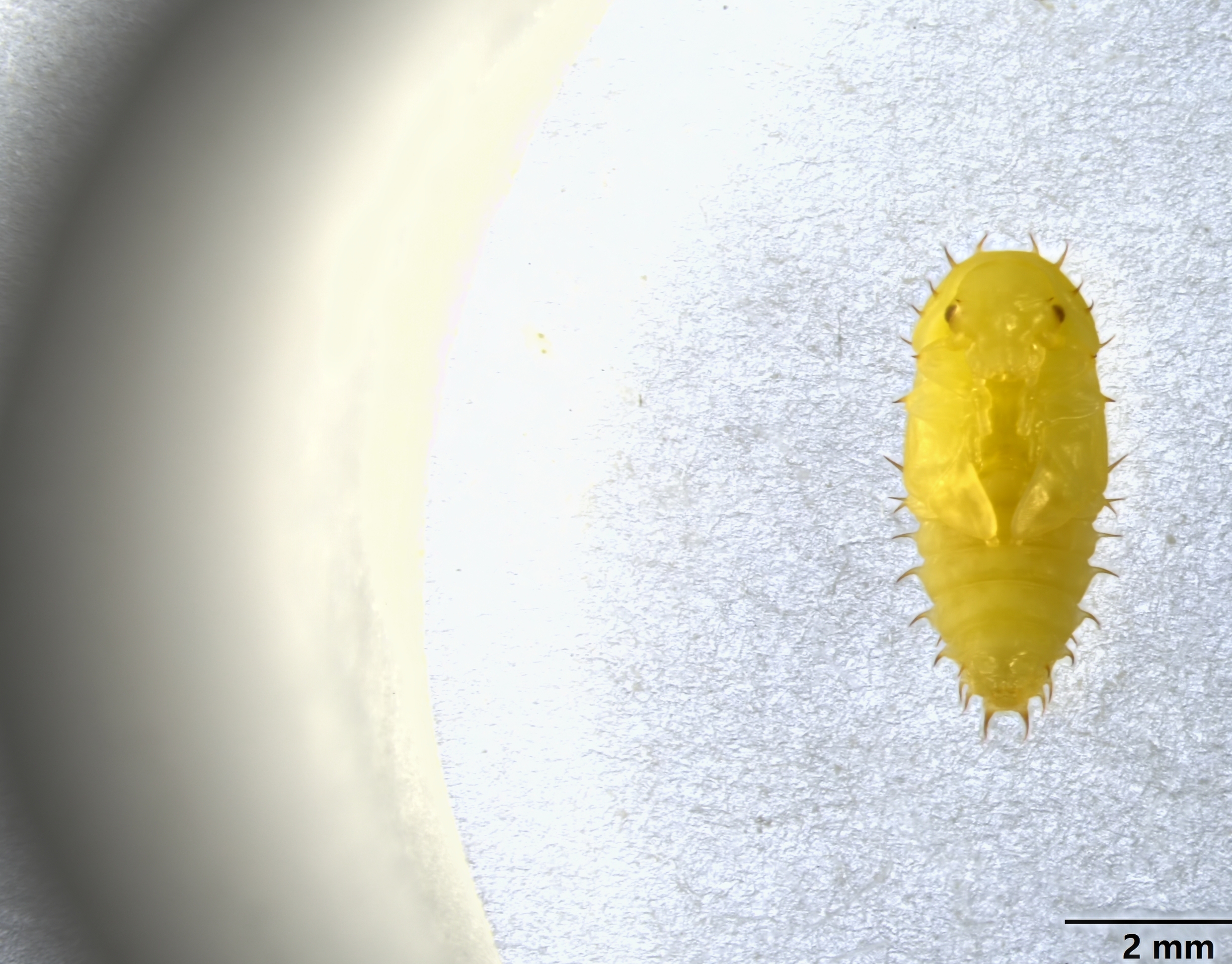

Supplement: Supplementary file 1 [file insects-17-00344-s001.zip › Experimental Data on Urophorus humeralis Nails/Figure/ pupa / pupa .jpg]

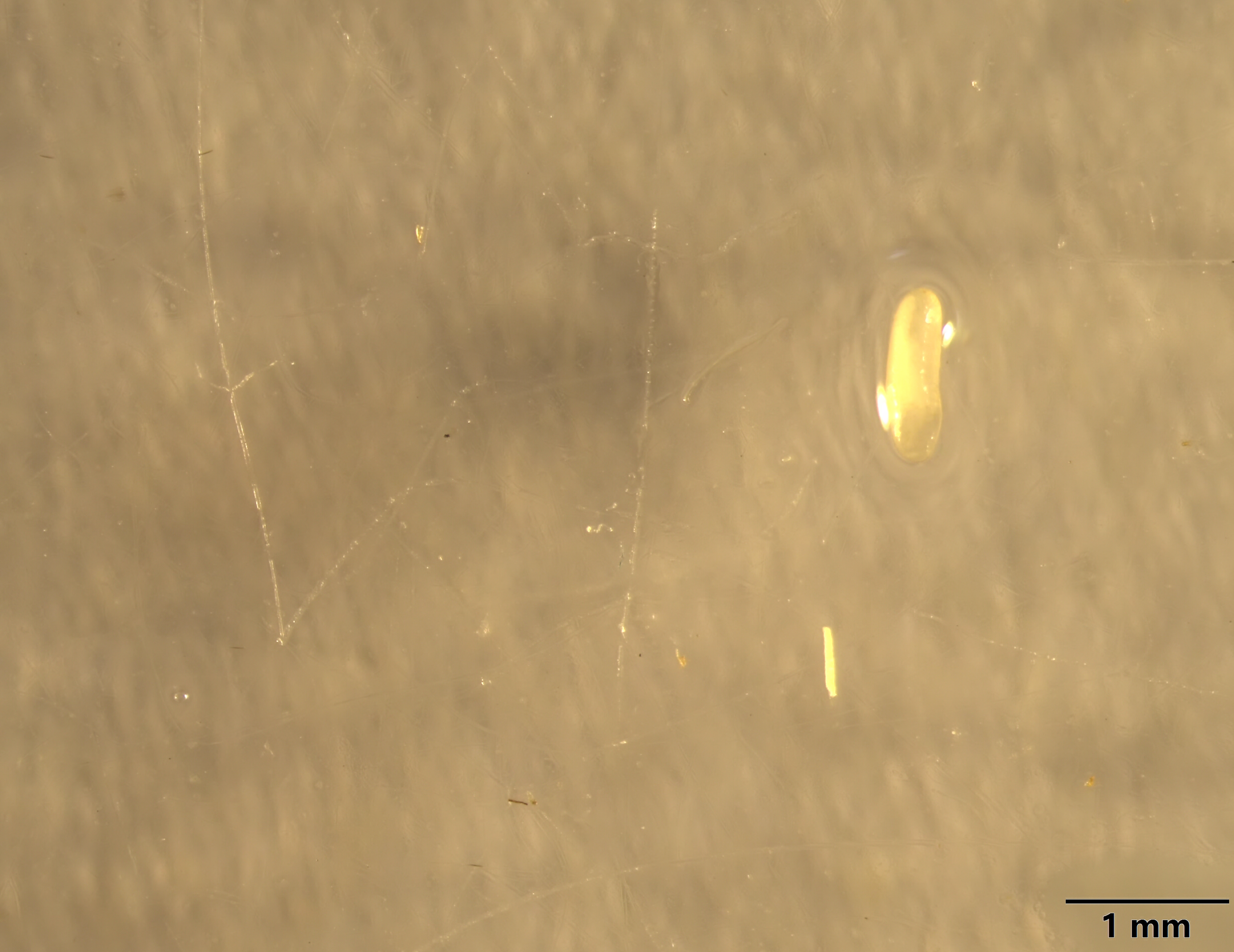

Supplement: Supplementary file 1 [file insects-17-00344-s001.zip › Experimental Data on Urophorus humeralis Nails/Figure/egg/egg.tif]

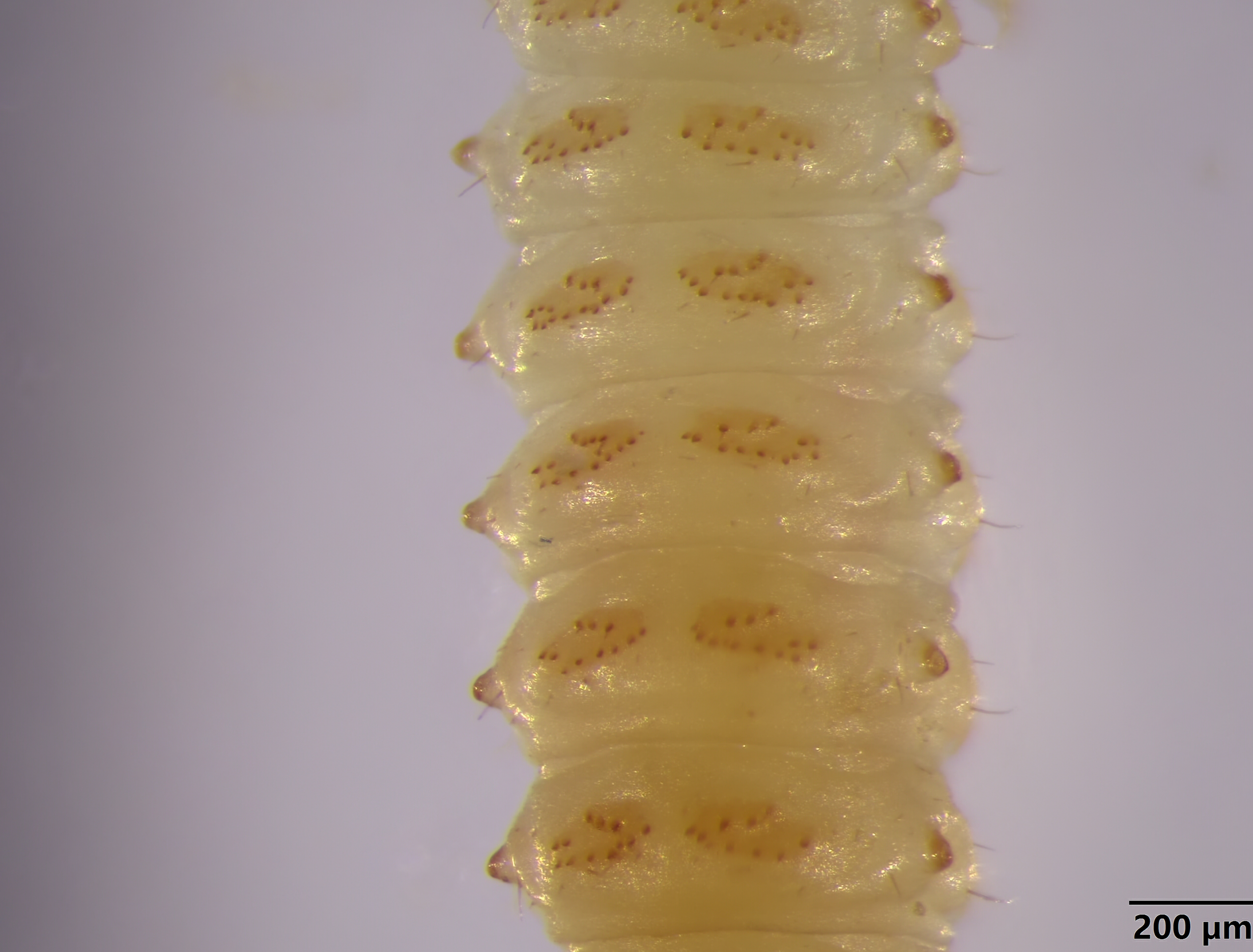

Supplement: Supplementary file 1 [file insects-17-00344-s001.zip › Experimental Data on Urophorus humeralis Nails/Figure/first-instar larvae/Abdominal segments displaying dorsal ossicles.tif]

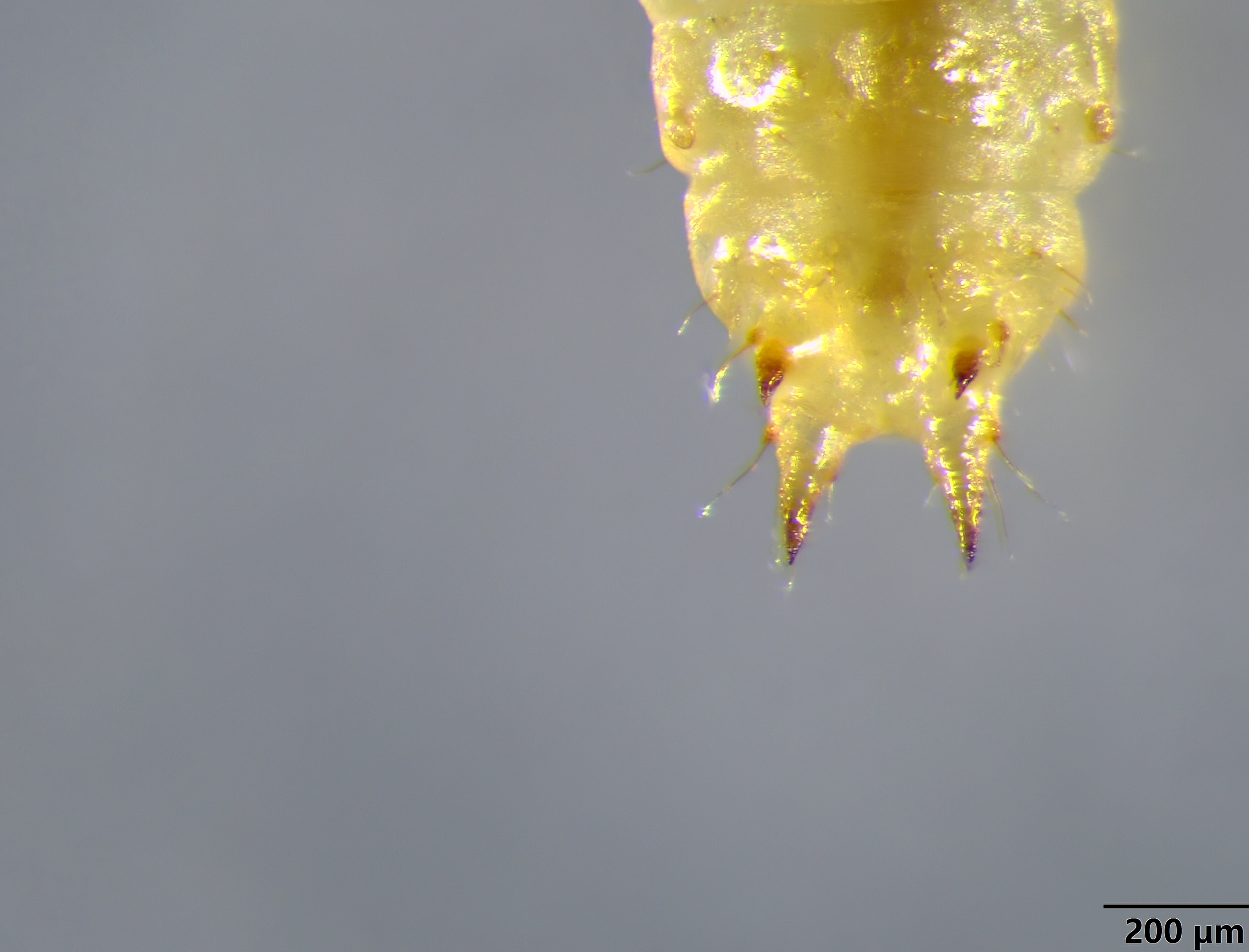

Supplement: Supplementary file 1 [file insects-17-00344-s001.zip › Experimental Data on Urophorus humeralis Nails/Figure/first-instar larvae/caudal processes.jpg]

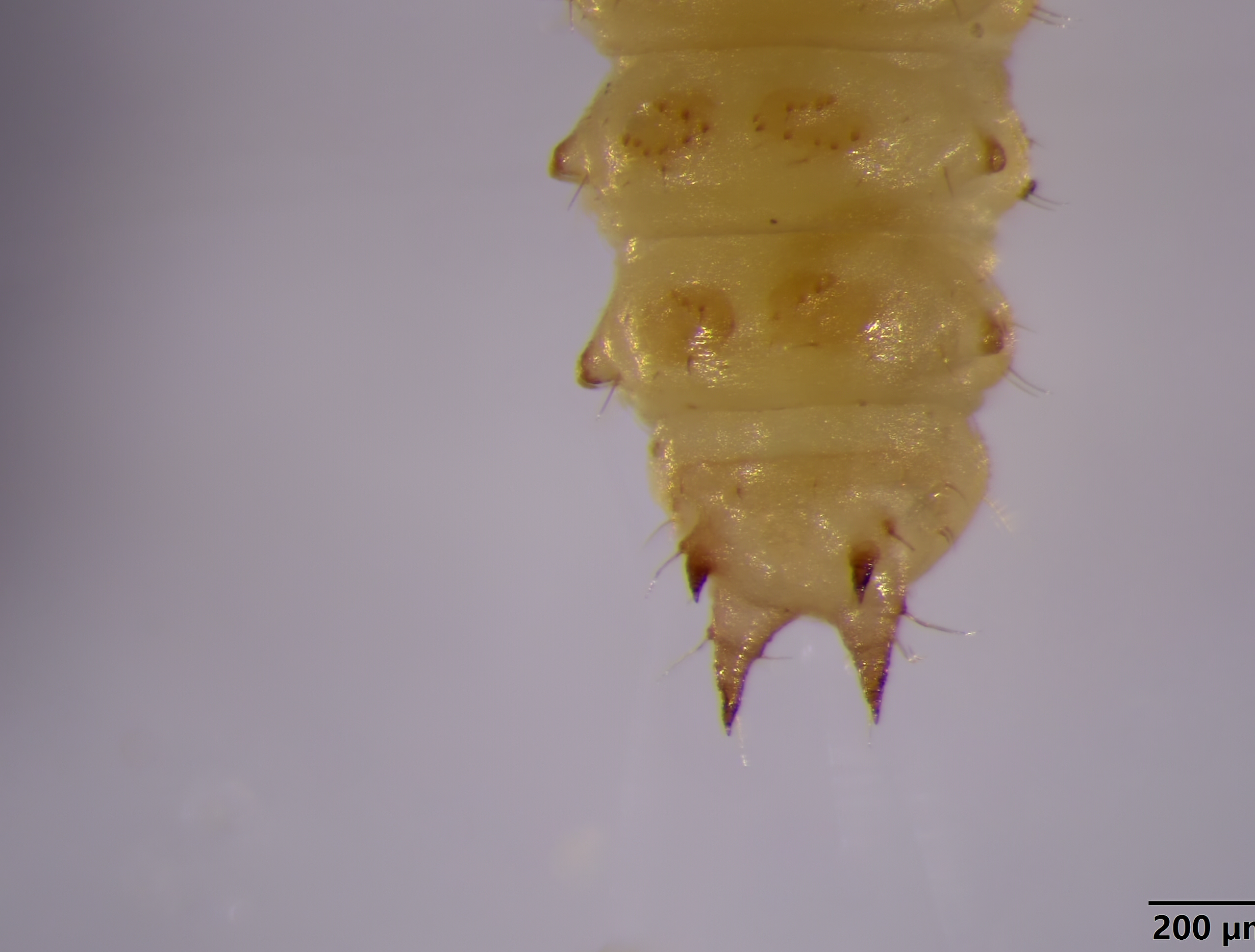

Supplement: Supplementary file 1 [file insects-17-00344-s001.zip › Experimental Data on Urophorus humeralis Nails/Figure/first-instar larvae/caudal processes.jpg2.tif]

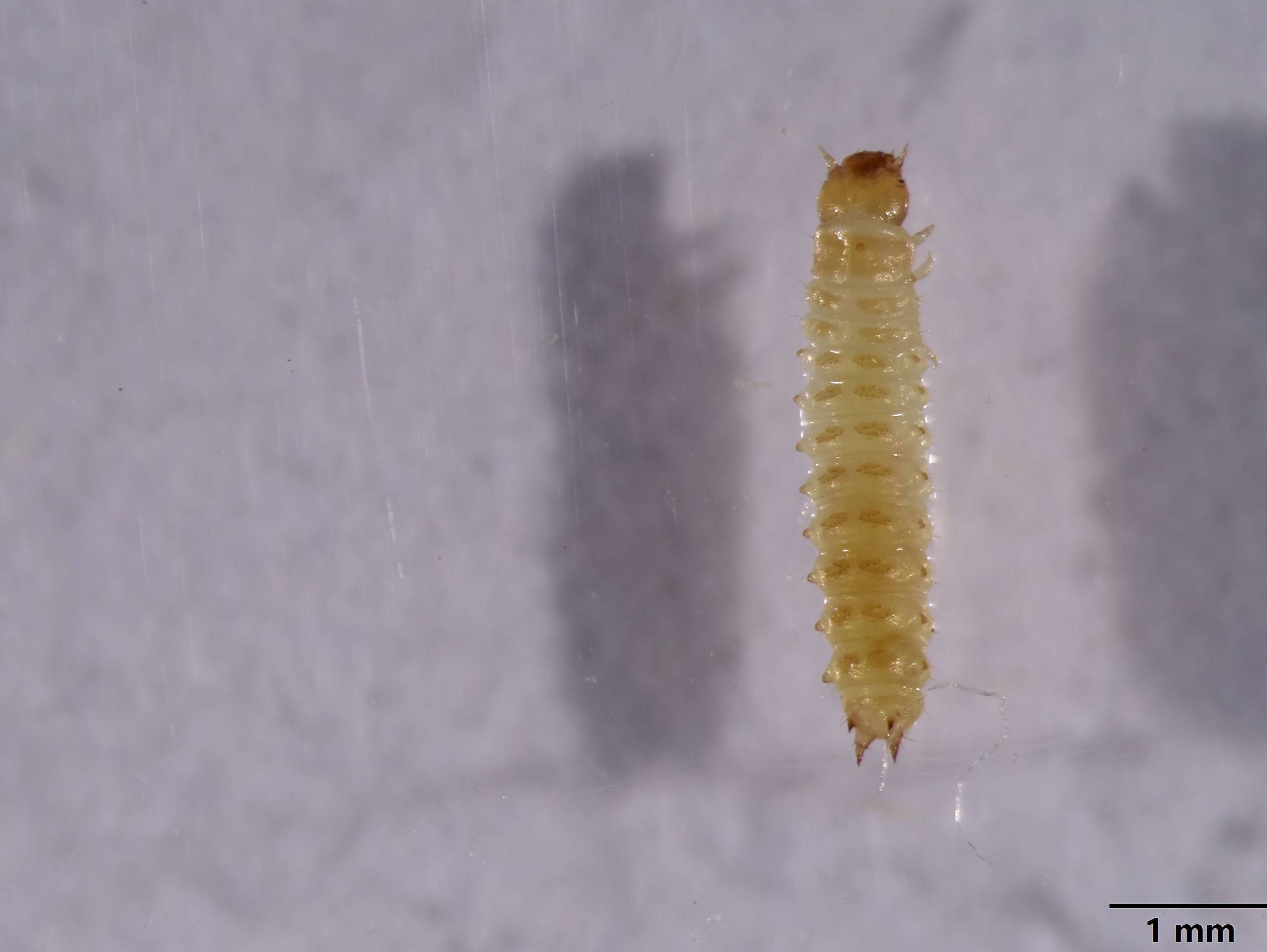

Supplement: Supplementary file 1 [file insects-17-00344-s001.zip › Experimental Data on Urophorus humeralis Nails/Figure/first-instar larvae/dorsal view.tif]

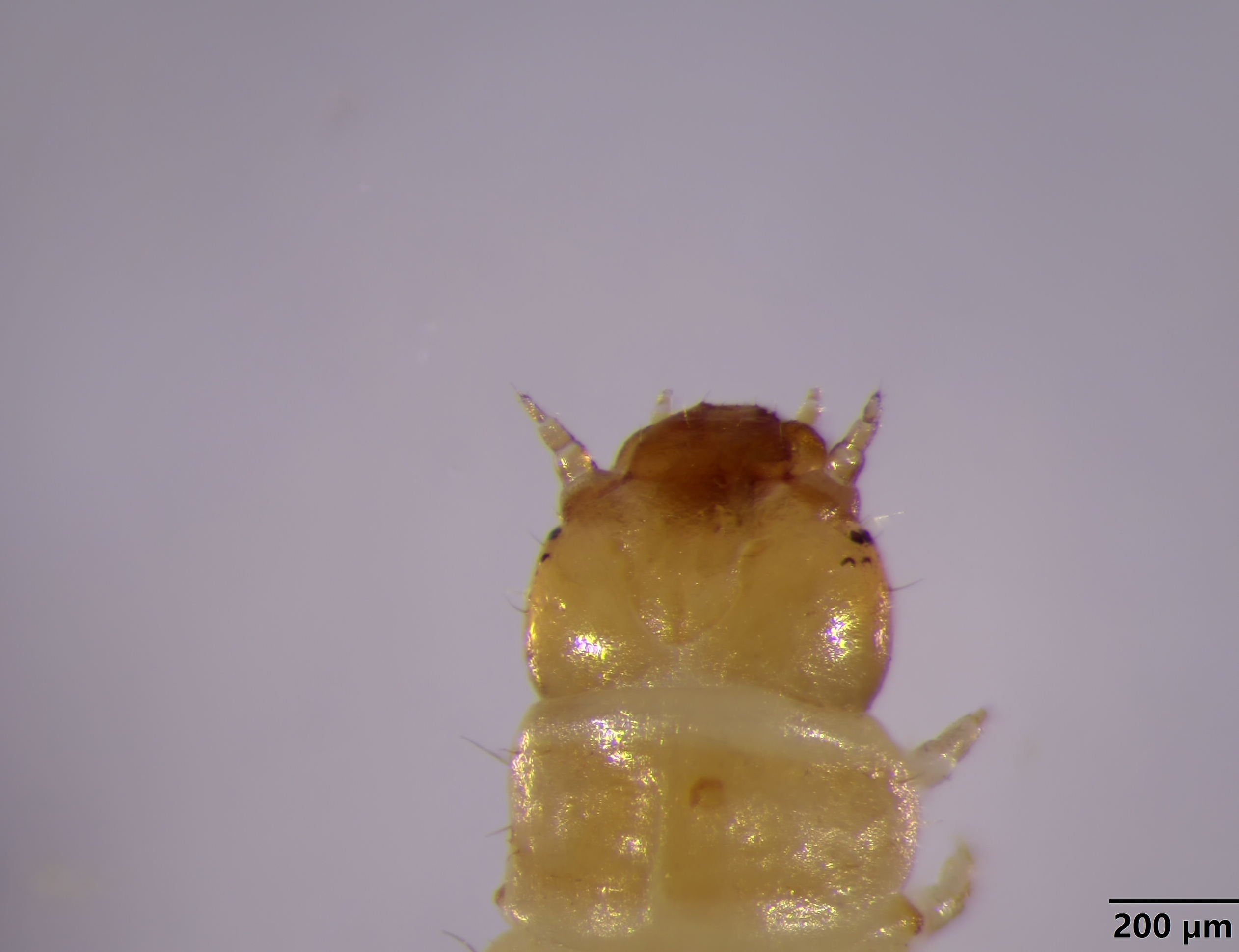

Supplement: Supplementary file 1 [file insects-17-00344-s001.zip › Experimental Data on Urophorus humeralis Nails/Figure/first-instar larvae/head capsule.tif]

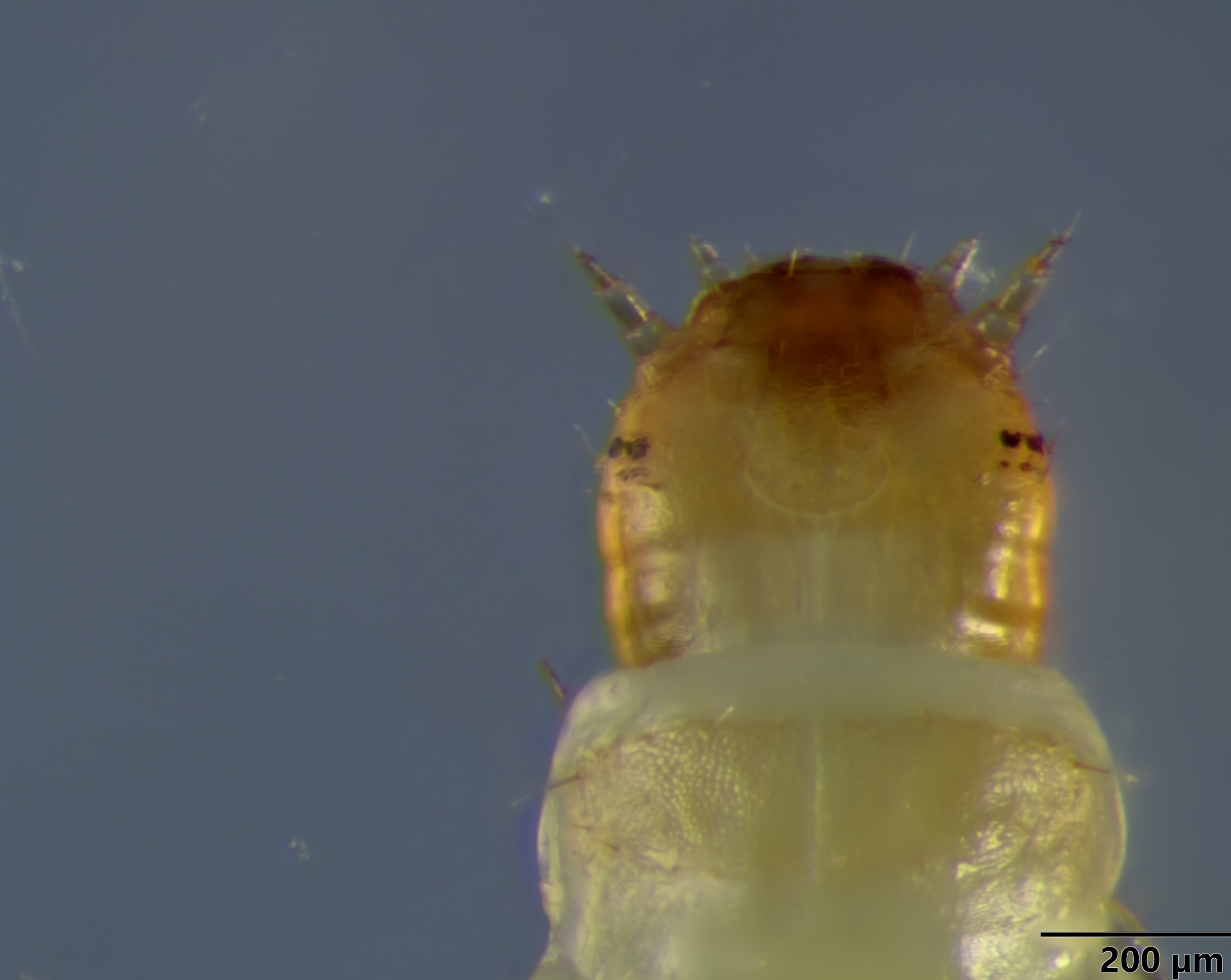

Supplement: Supplementary file 1 [file insects-17-00344-s001.zip › Experimental Data on Urophorus humeralis Nails/Figure/first-instar larvae/Head.jpg]

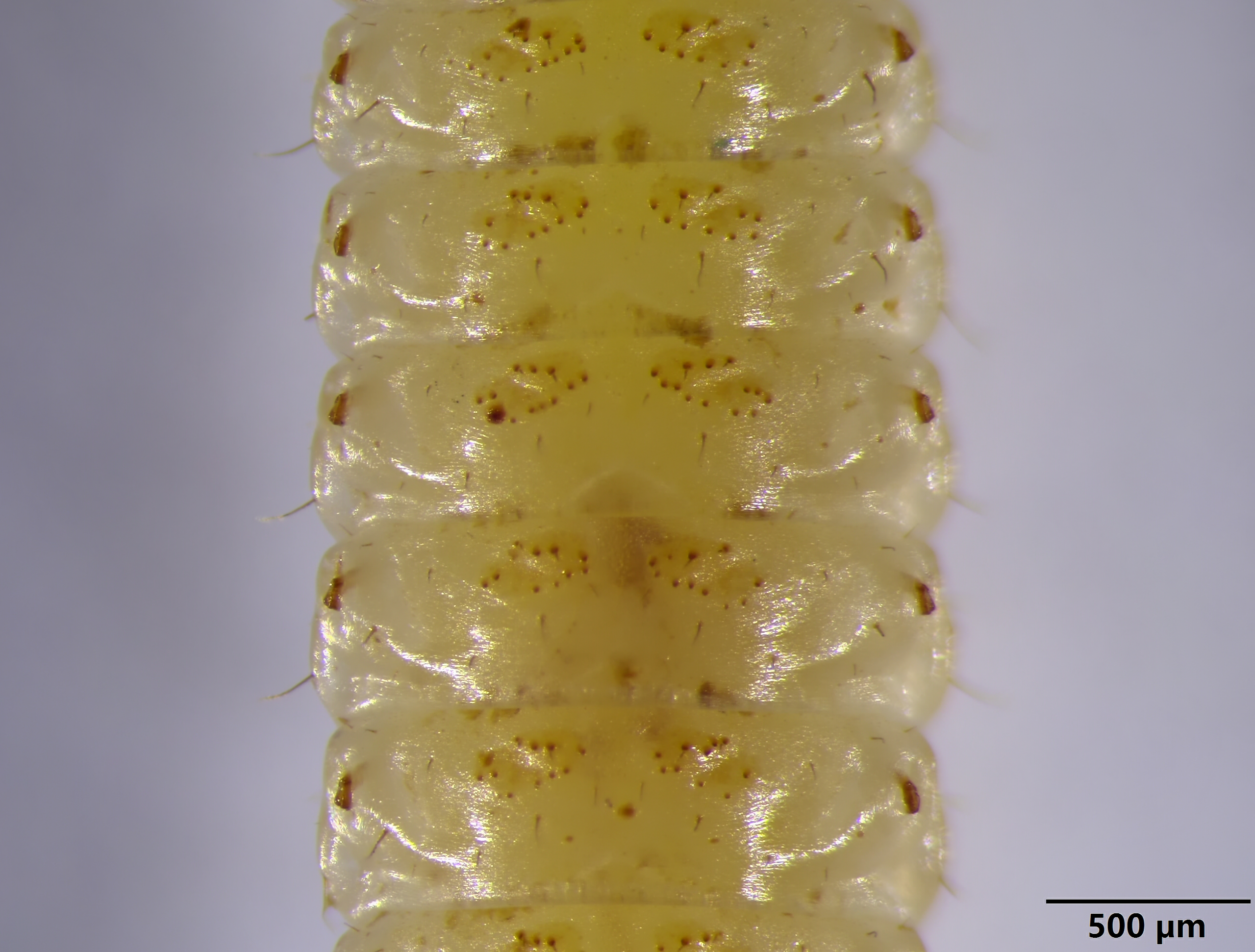

Supplement: Supplementary file 1 [file insects-17-00344-s001.zip › Experimental Data on Urophorus humeralis Nails/Figure/second-instar larvae/Abdominal segments displaying dorsal ossicles.tif]

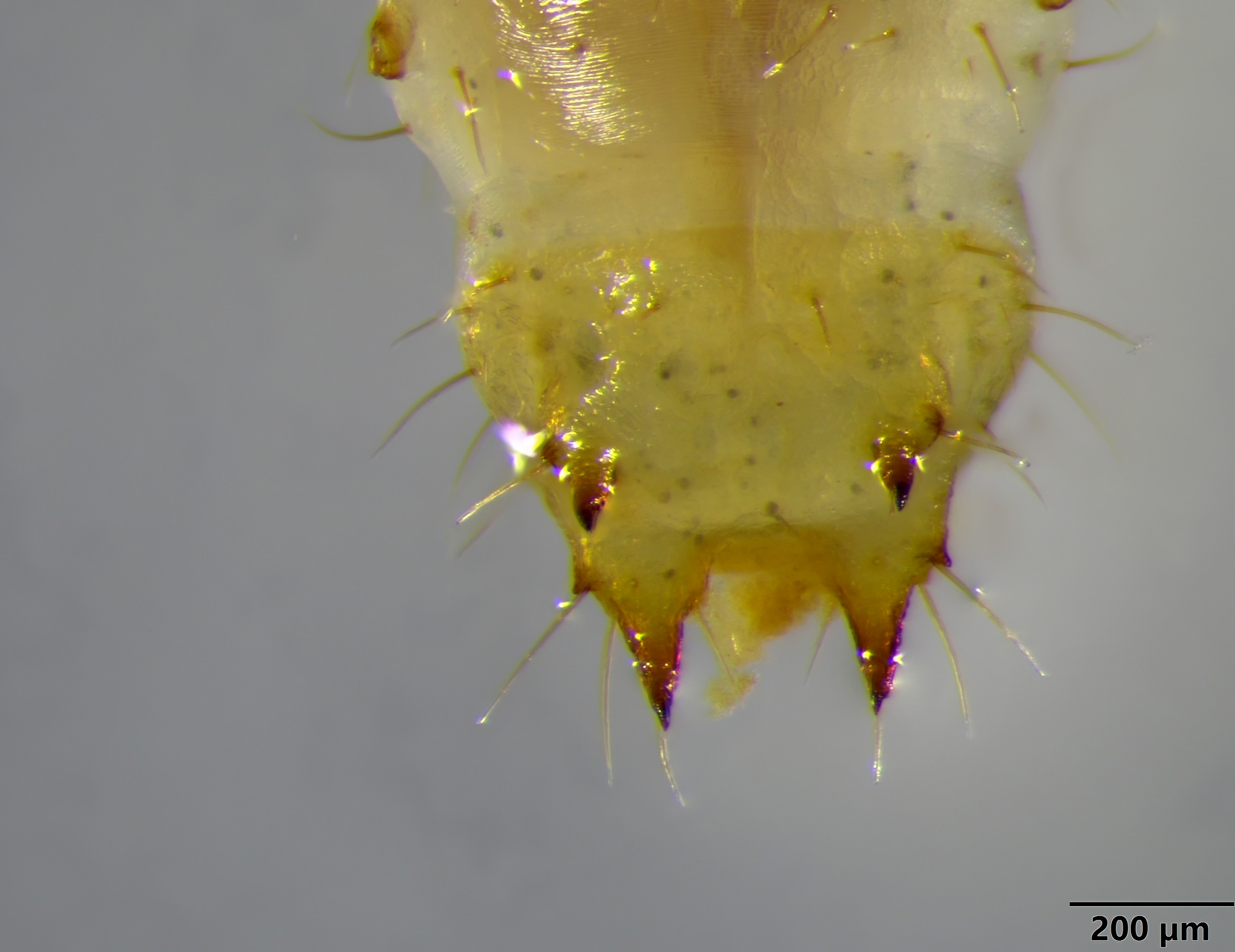

Supplement: Supplementary file 1 [file insects-17-00344-s001.zip › Experimental Data on Urophorus humeralis Nails/Figure/second-instar larvae/caudal processes.jpg]

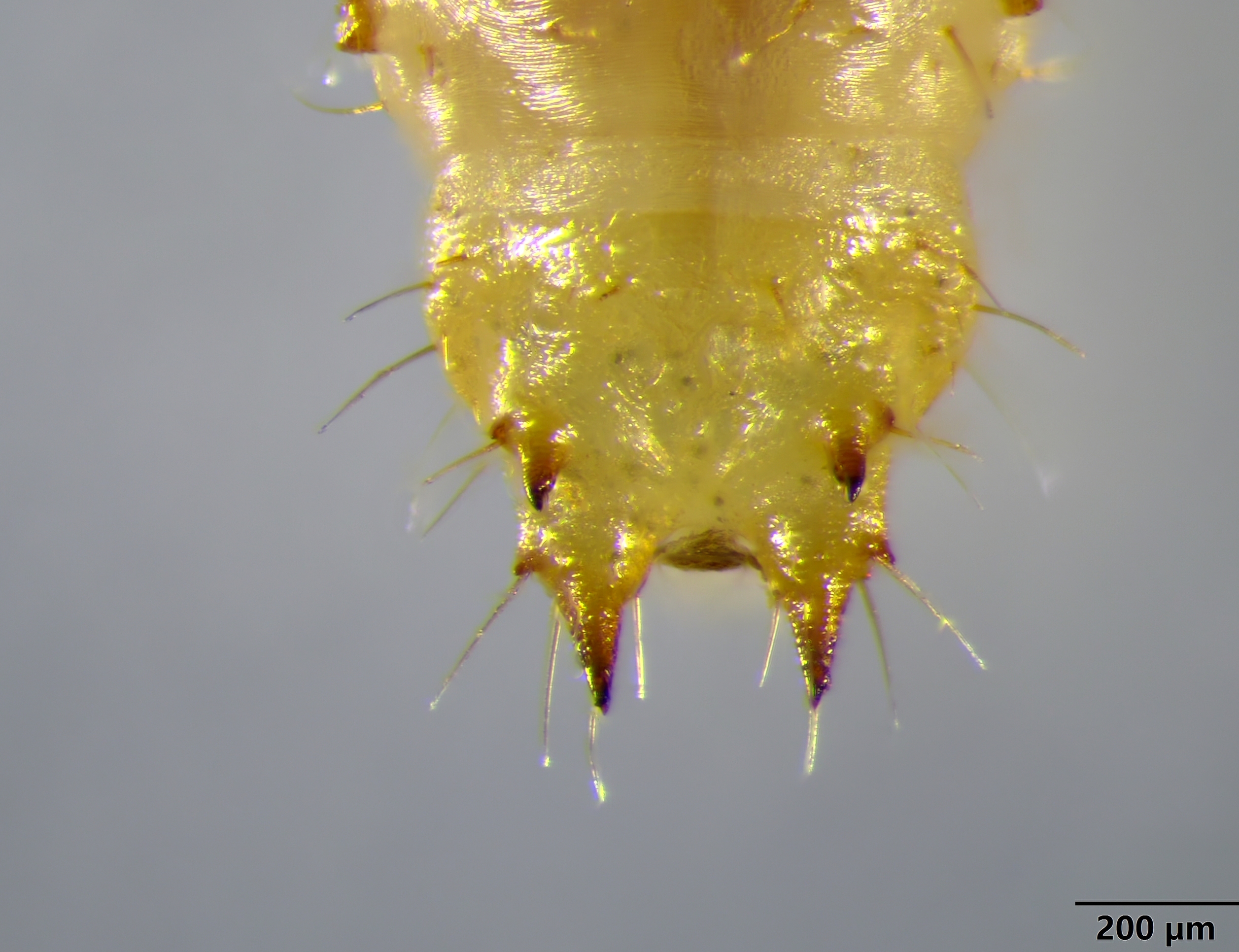

Supplement: Supplementary file 1 [file insects-17-00344-s001.zip › Experimental Data on Urophorus humeralis Nails/Figure/second-instar larvae/caudal processes2.jpg]

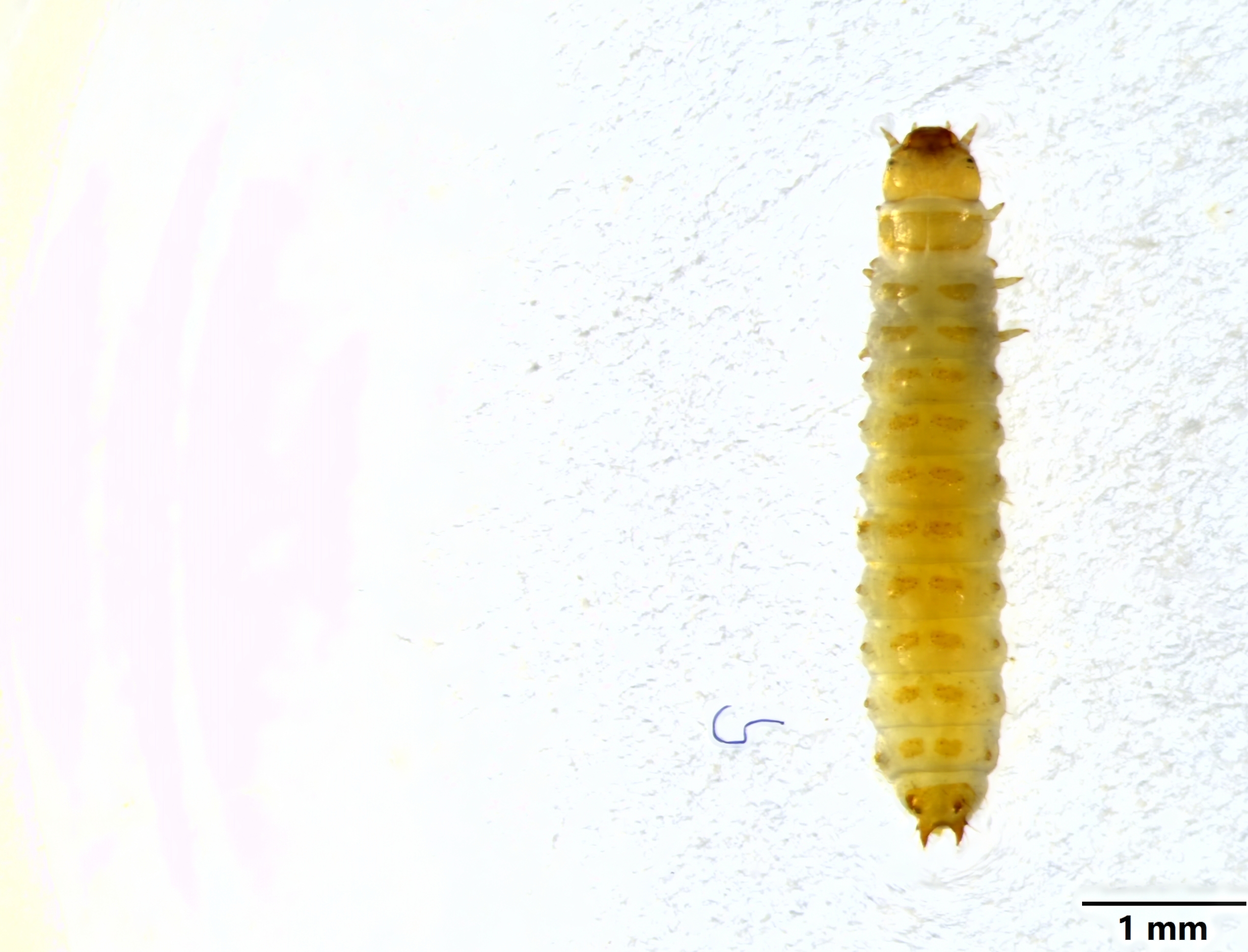

Supplement: Supplementary file 1 [file insects-17-00344-s001.zip › Experimental Data on Urophorus humeralis Nails/Figure/second-instar larvae/dorsal view.jpg]

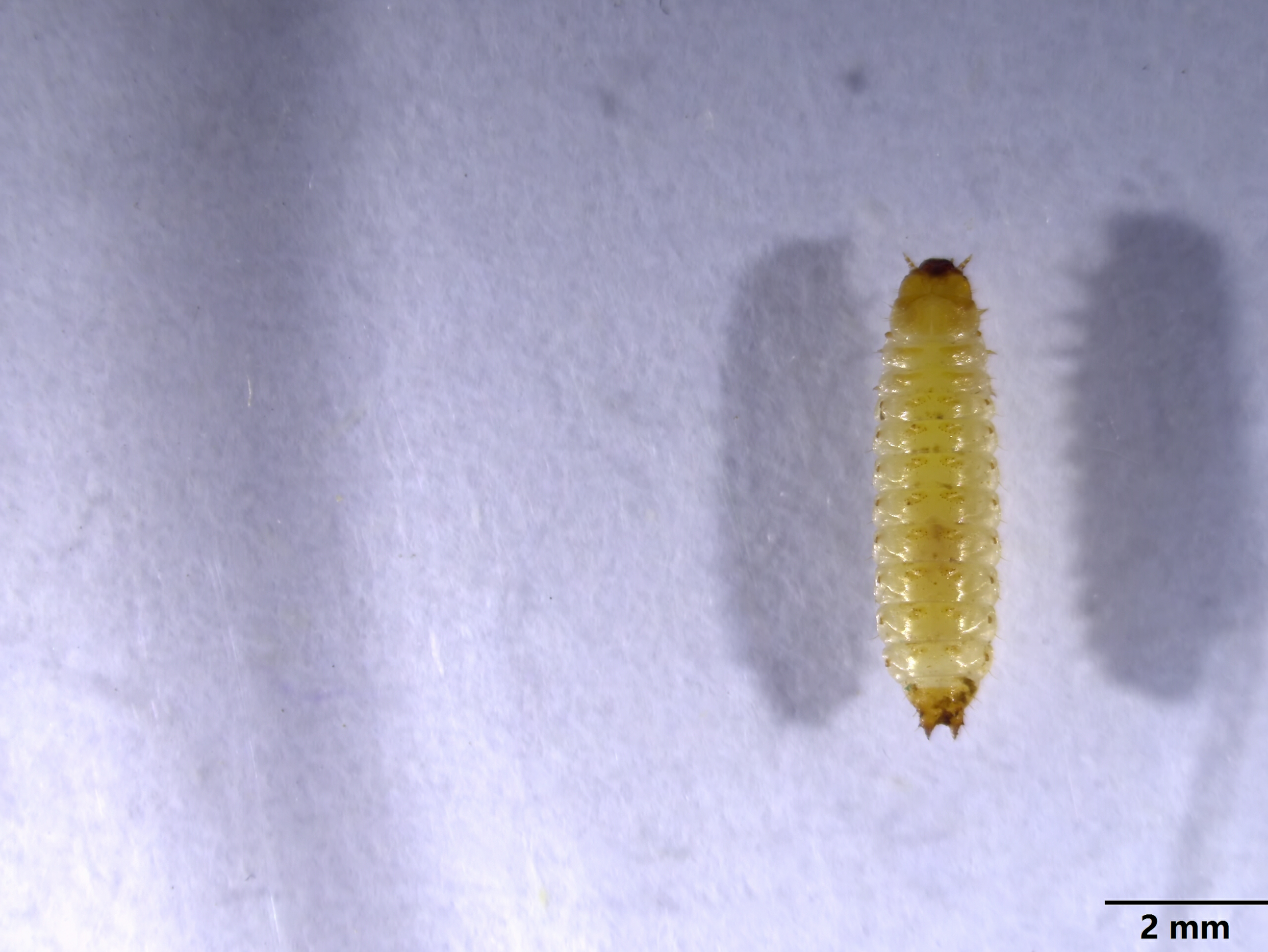

Supplement: Supplementary file 1 [file insects-17-00344-s001.zip › Experimental Data on Urophorus humeralis Nails/Figure/second-instar larvae/dorsal view2.tif]

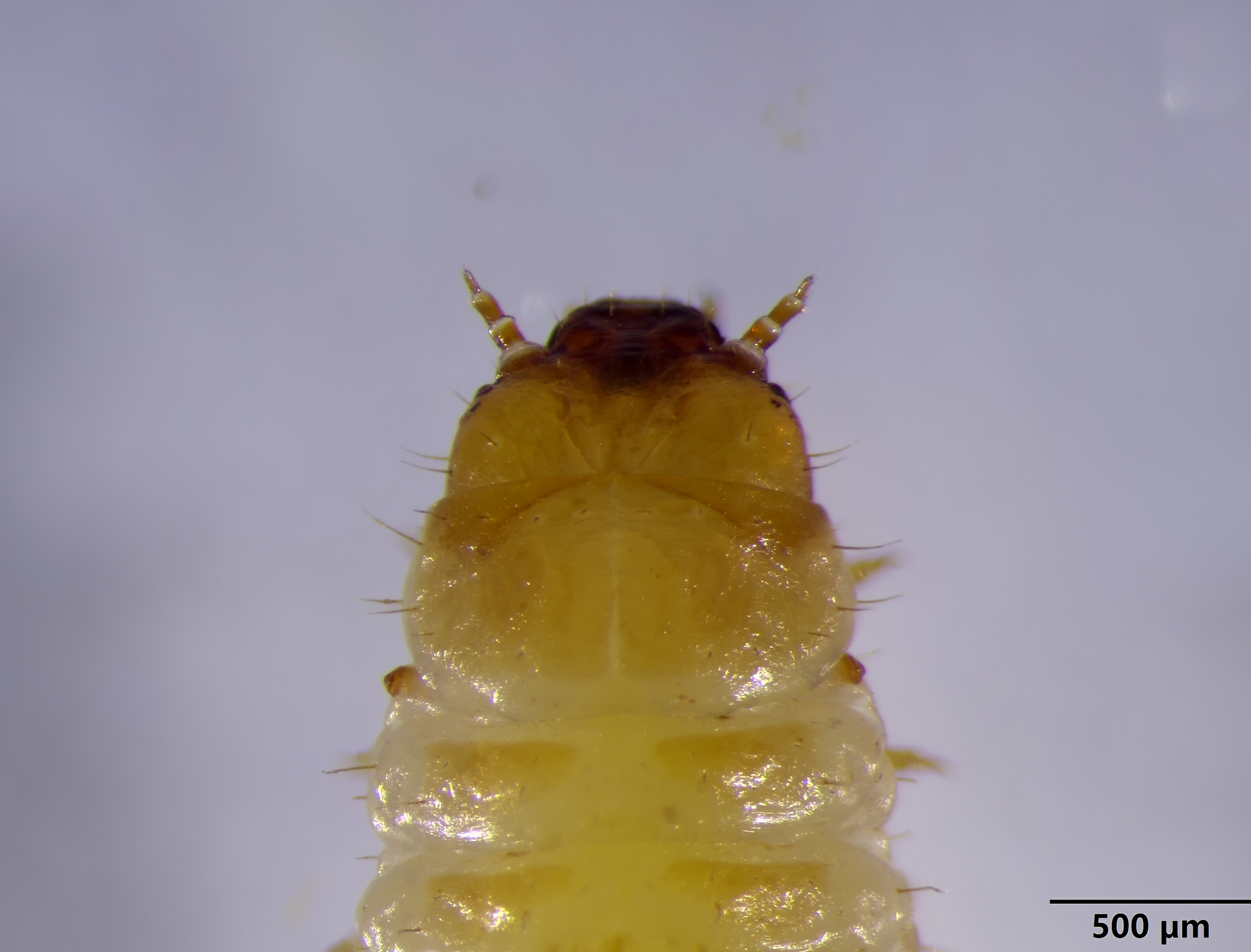

Supplement: Supplementary file 1 [file insects-17-00344-s001.zip › Experimental Data on Urophorus humeralis Nails/Figure/second-instar larvae/head.tif]

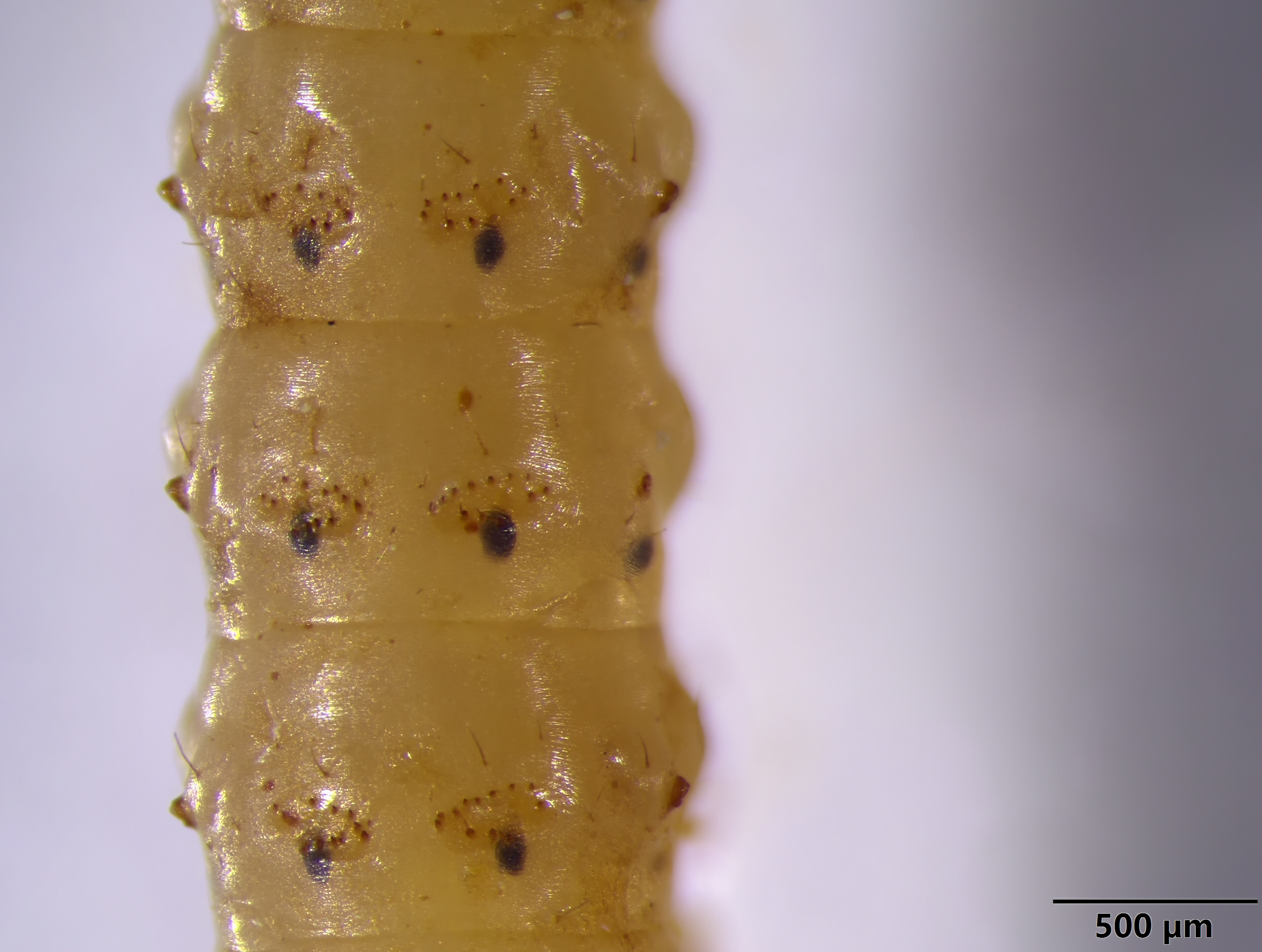

Supplement: Supplementary file 1 [file insects-17-00344-s001.zip › Experimental Data on Urophorus humeralis Nails/Figure/third-instar larvae/Abdominal segments displaying dorsal ossicles.tif]

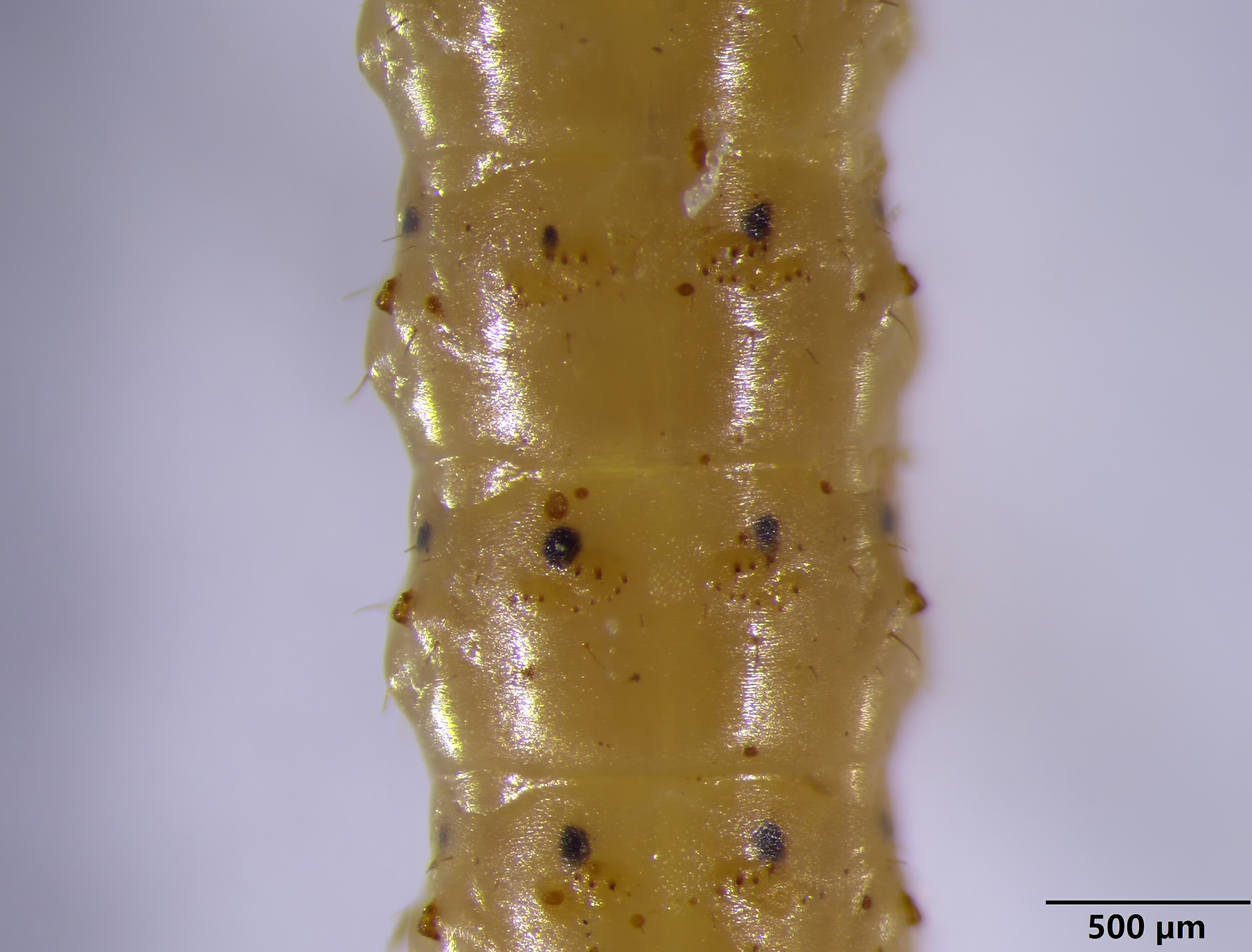

Supplement: Supplementary file 1 [file insects-17-00344-s001.zip › Experimental Data on Urophorus humeralis Nails/Figure/third-instar larvae/Abdominal segments displaying dorsal ossicles2.tif]

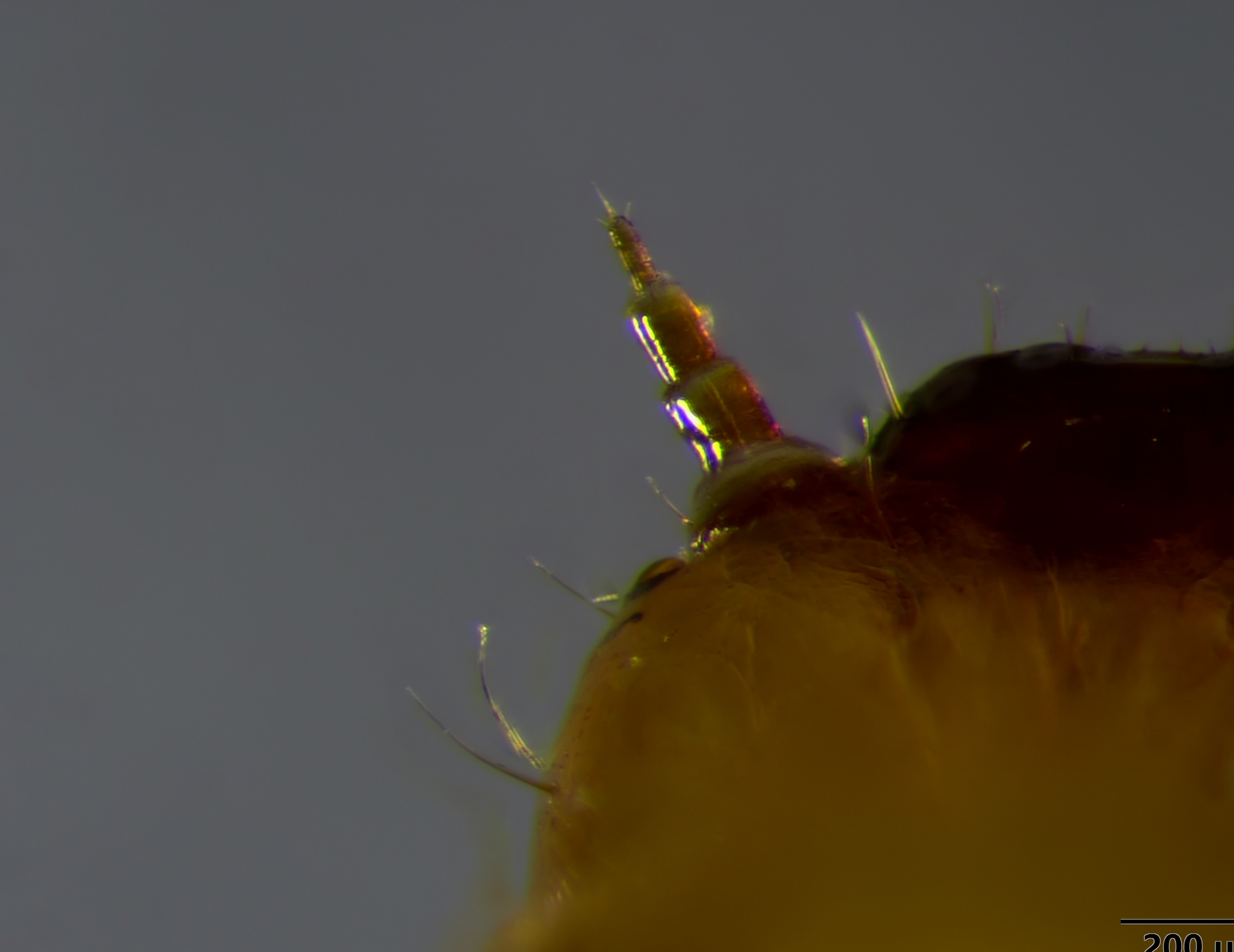

Supplement: Supplementary file 1 [file insects-17-00344-s001.zip › Experimental Data on Urophorus humeralis Nails/Figure/third-instar larvae/Antenna.jpg]

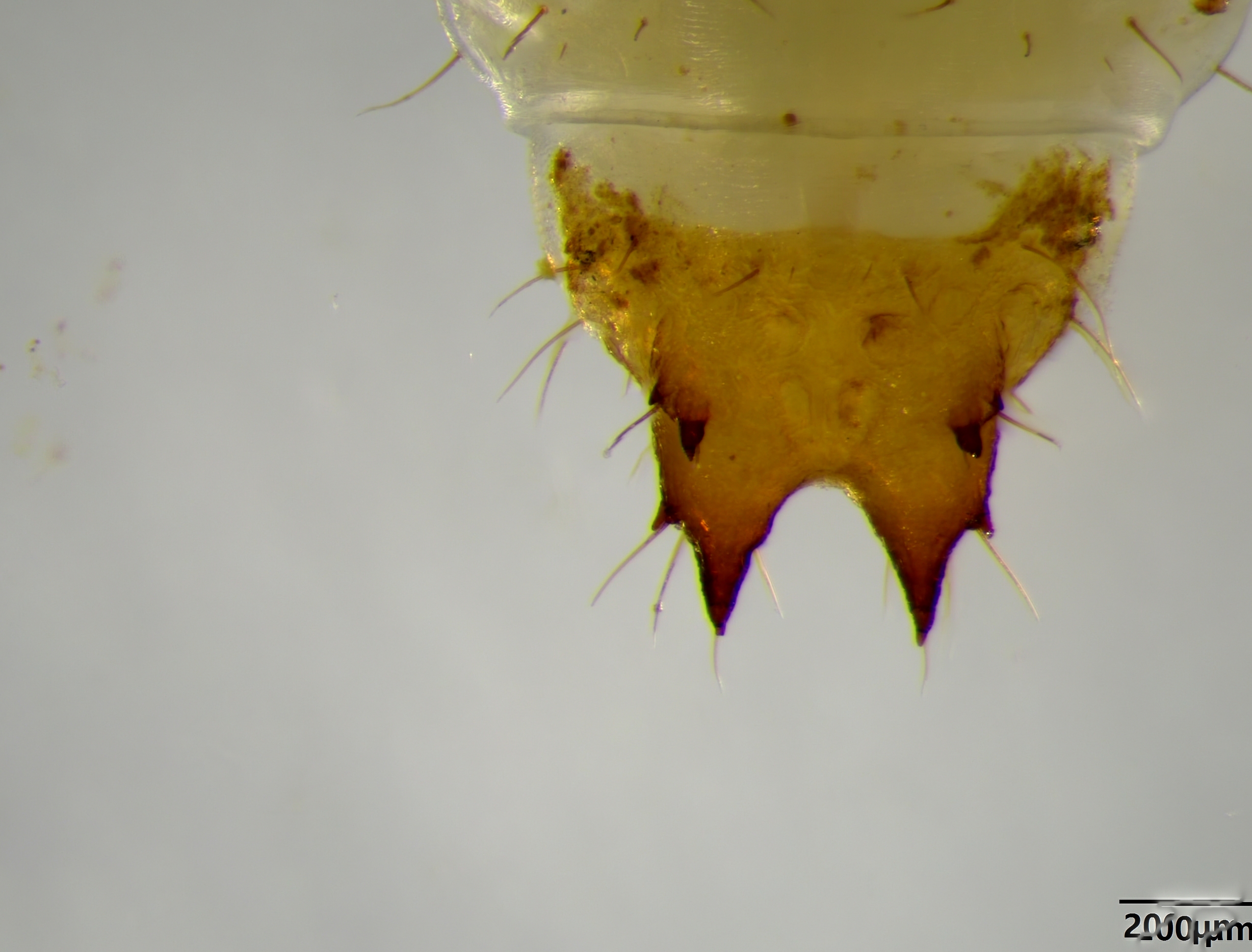

Supplement: Supplementary file 1 [file insects-17-00344-s001.zip › Experimental Data on Urophorus humeralis Nails/Figure/third-instar larvae/caudal processes.jpg]

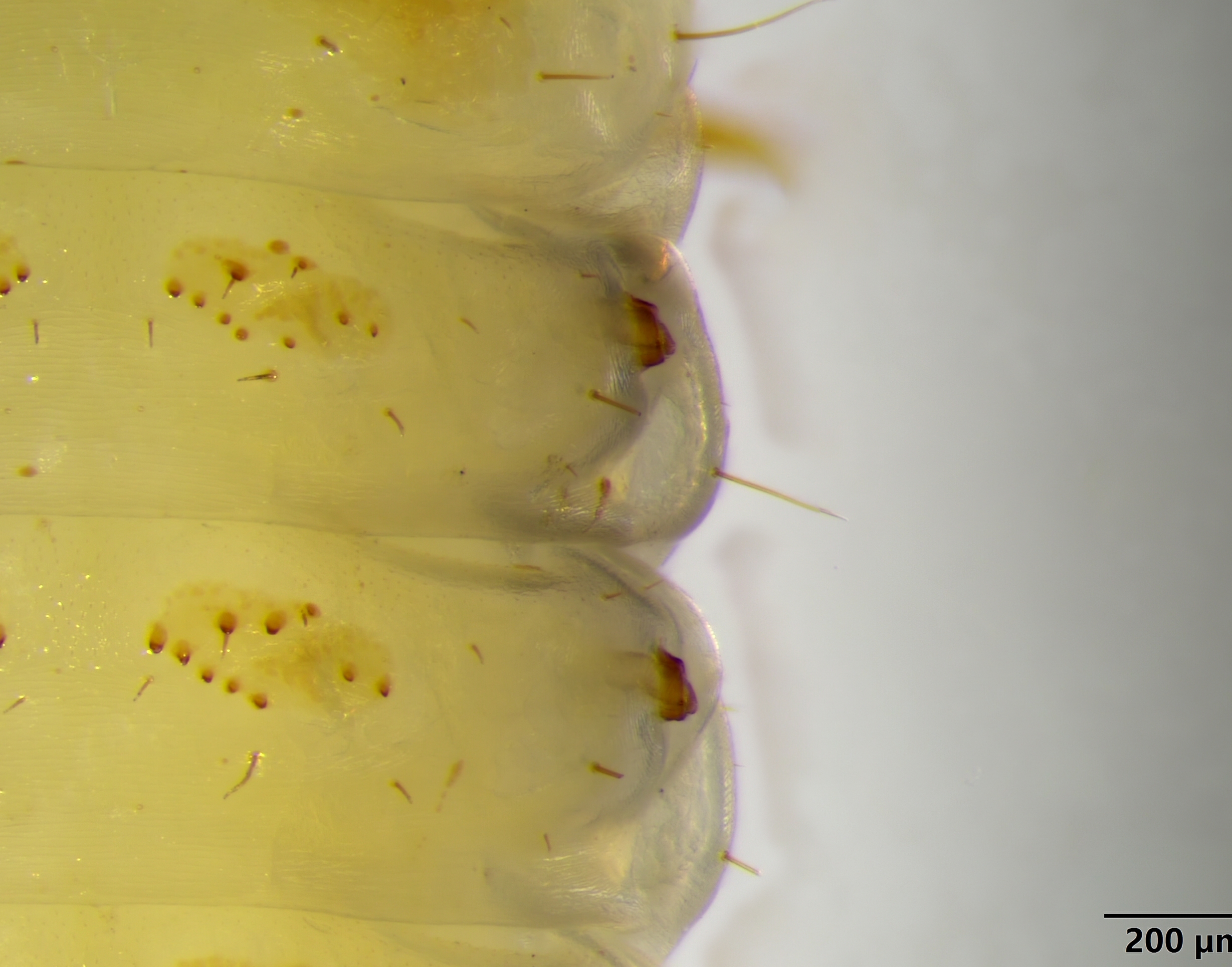

Supplement: Supplementary file 1 [file insects-17-00344-s001.zip › Experimental Data on Urophorus humeralis Nails/Figure/third-instar larvae/Dorsal .jpg]

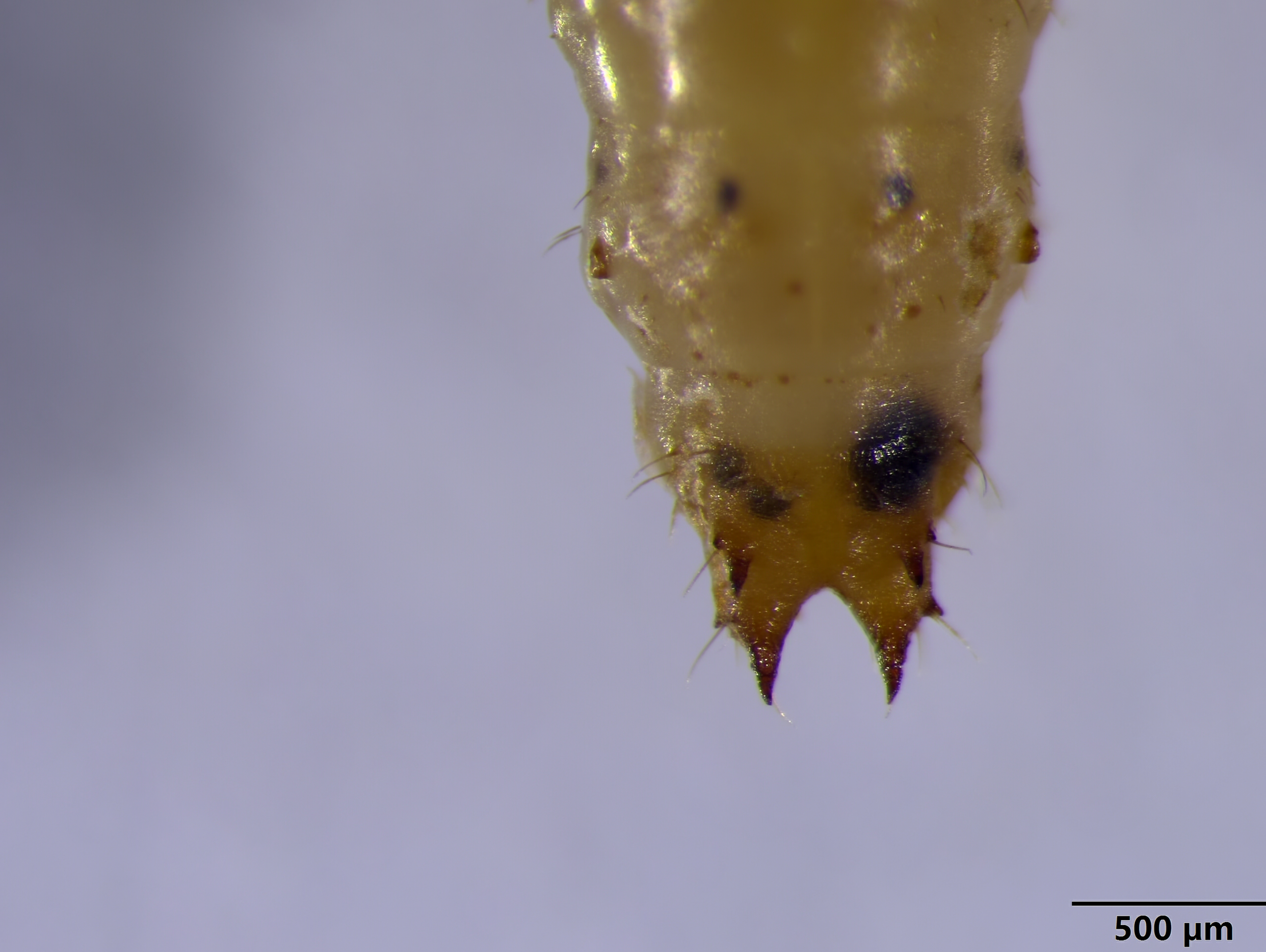

Supplement: Supplementary file 1 [file insects-17-00344-s001.zip › Experimental Data on Urophorus humeralis Nails/Figure/third-instar larvae/dorsal surface of the caudal process.tif]

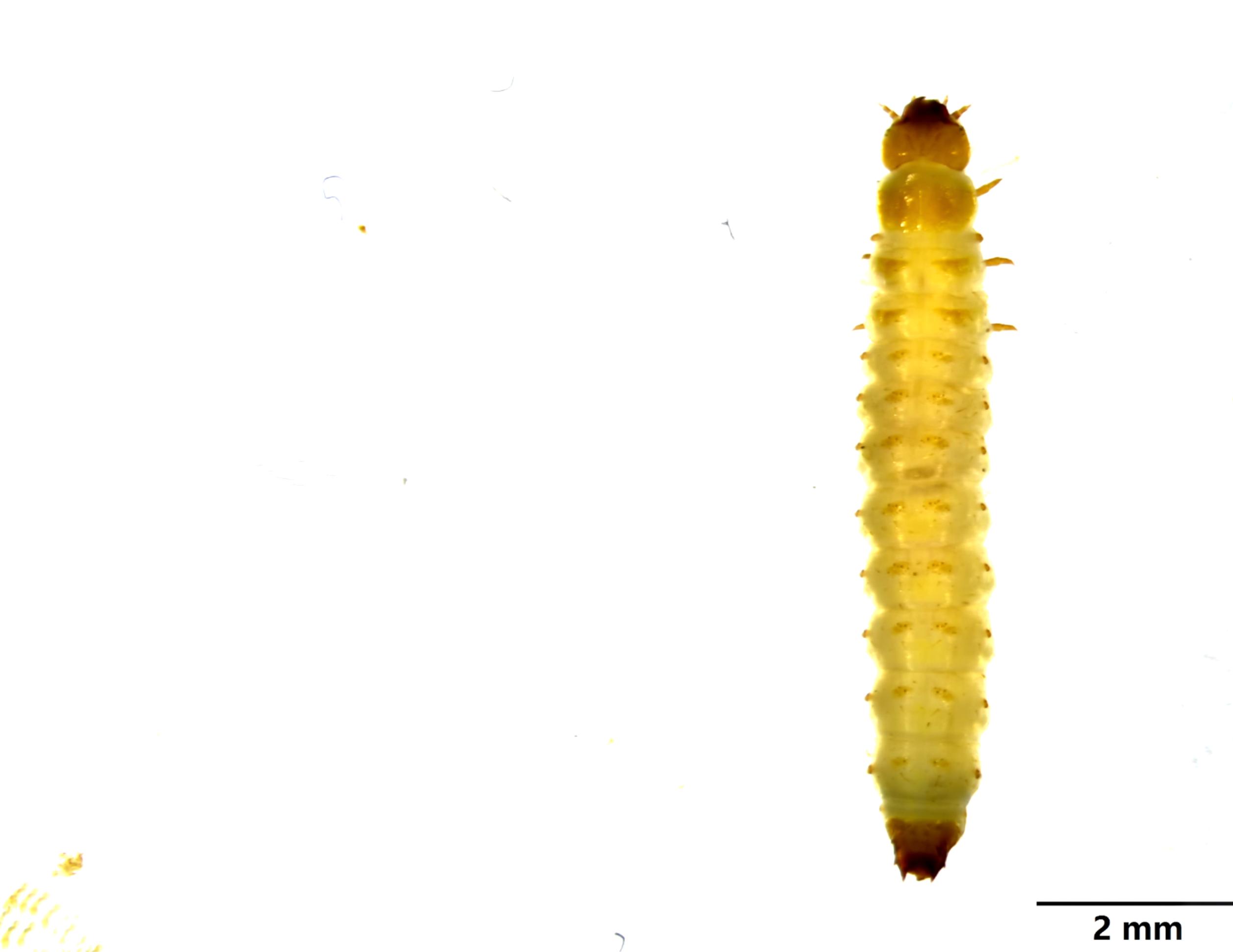

Supplement: Supplementary file 1 [file insects-17-00344-s001.zip › Experimental Data on Urophorus humeralis Nails/Figure/third-instar larvae/dorsal view.jpg]

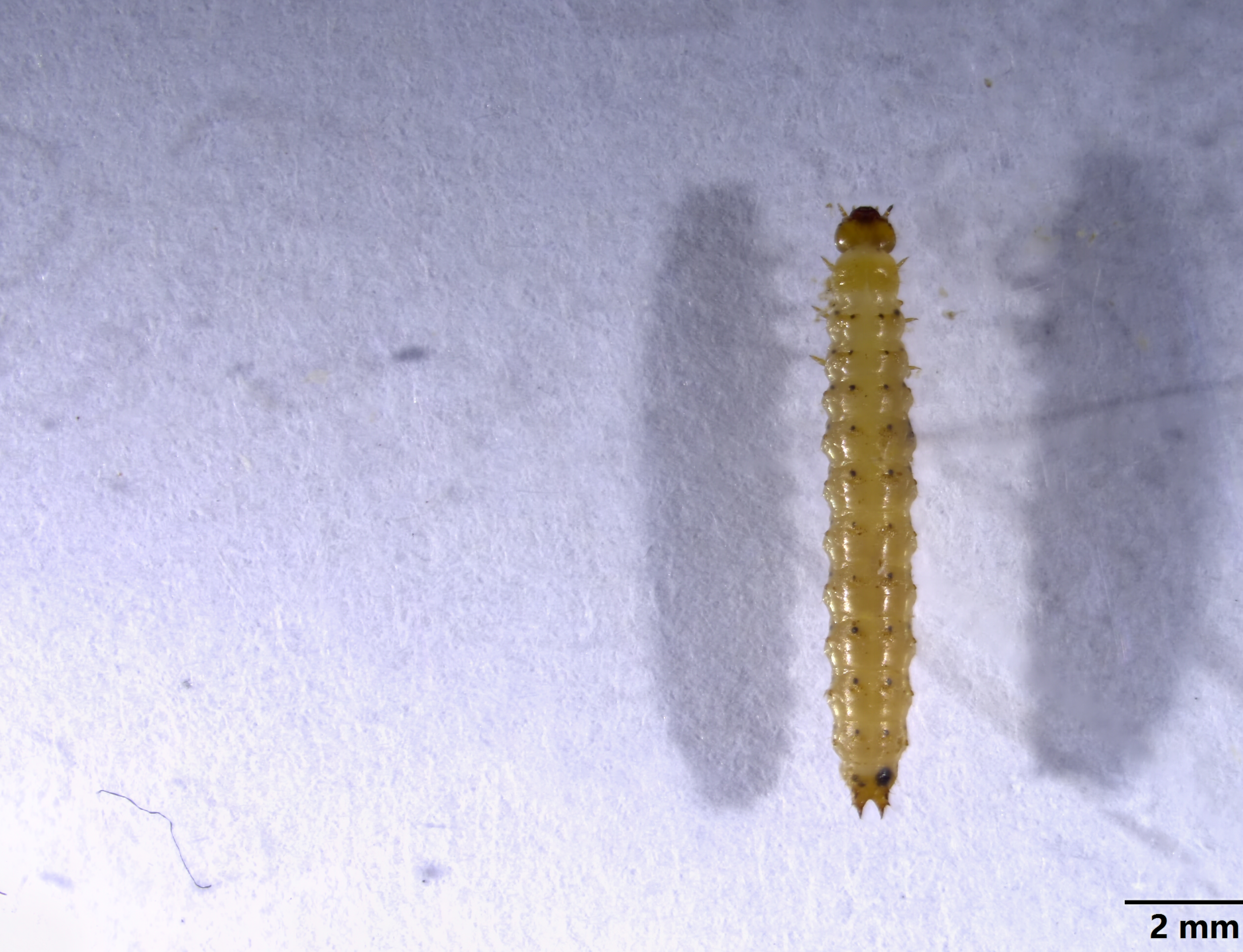

Supplement: Supplementary file 1 [file insects-17-00344-s001.zip › Experimental Data on Urophorus humeralis Nails/Figure/third-instar larvae/dorsal view.tif]

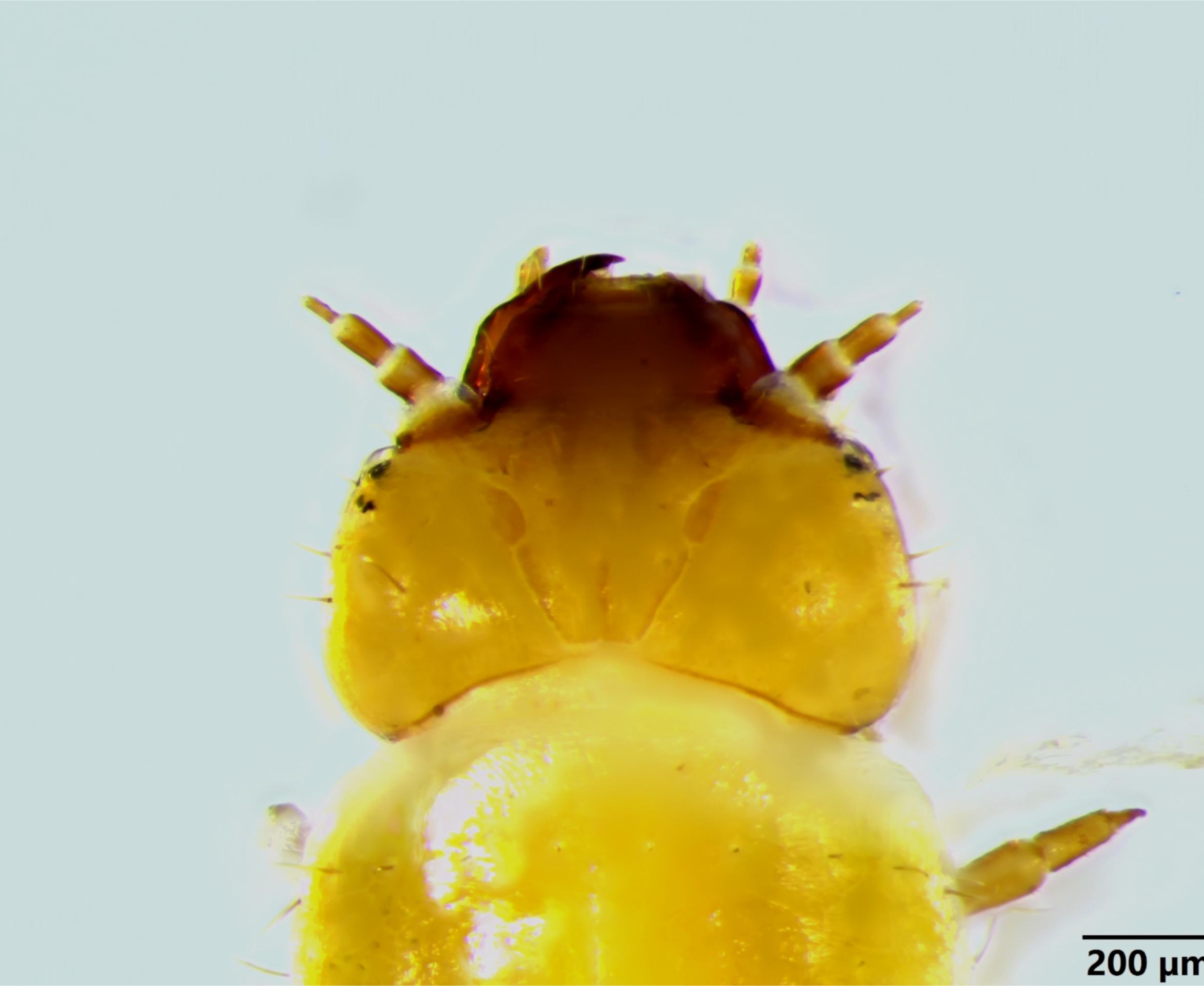

Supplement: Supplementary file 1 [file insects-17-00344-s001.zip › Experimental Data on Urophorus humeralis Nails/Figure/third-instar larvae/Head.jpg]

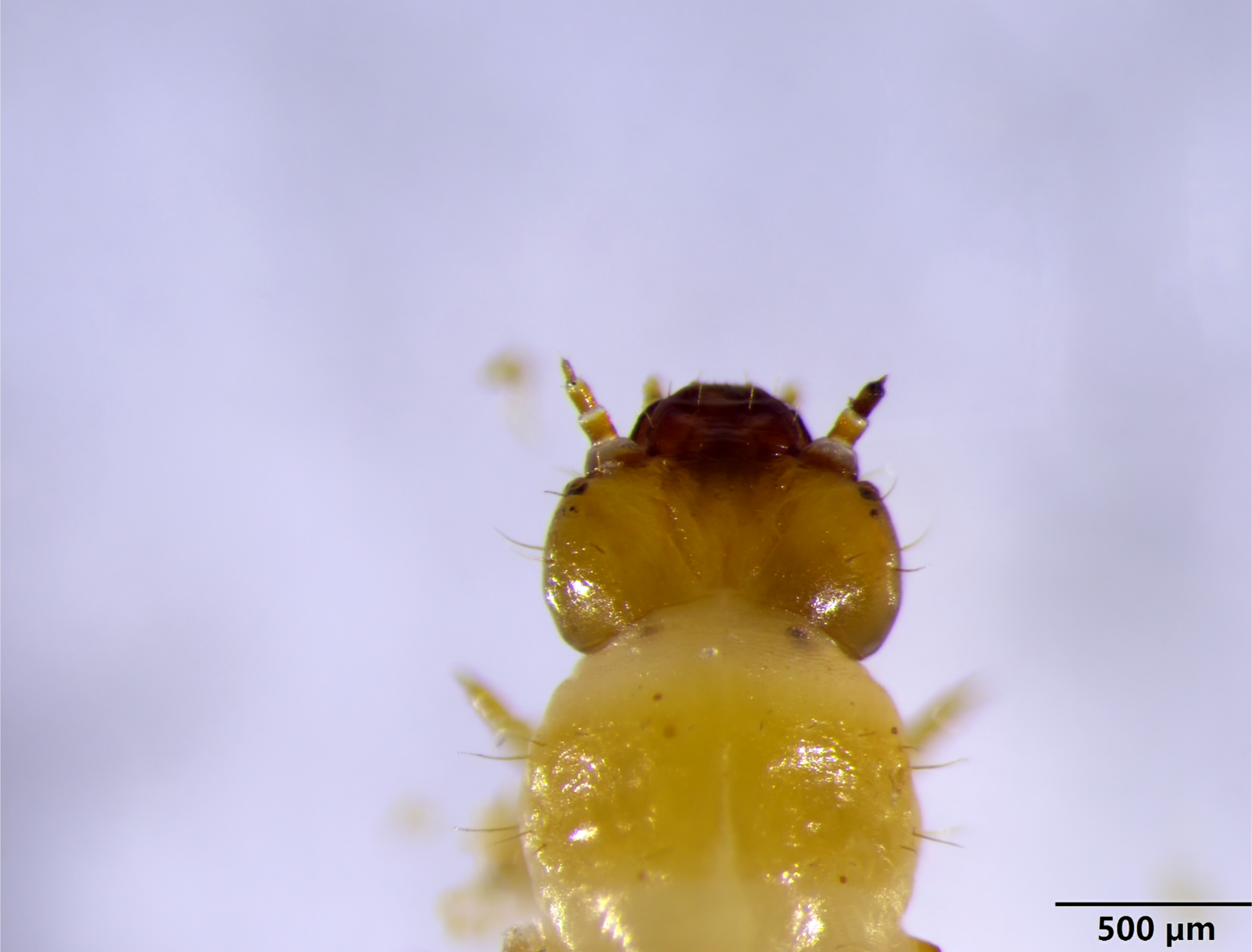

Supplement: Supplementary file 1 [file insects-17-00344-s001.zip › Experimental Data on Urophorus humeralis Nails/Figure/third-instar larvae/Head.png]

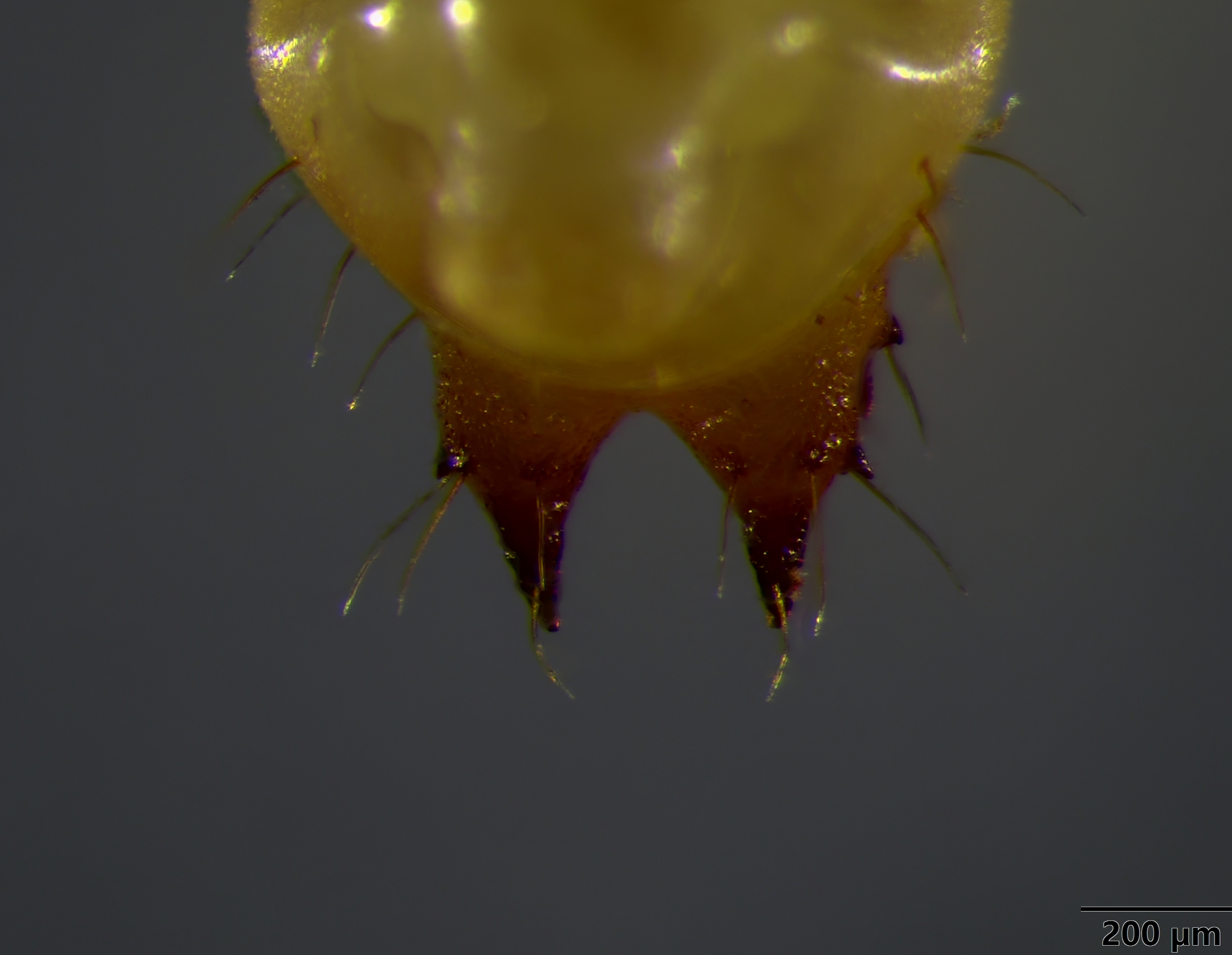

Supplement: Supplementary file 1 [file insects-17-00344-s001.zip › Experimental Data on Urophorus humeralis Nails/Figure/third-instar larvae/Ventral surface of the caudal process.jpg]

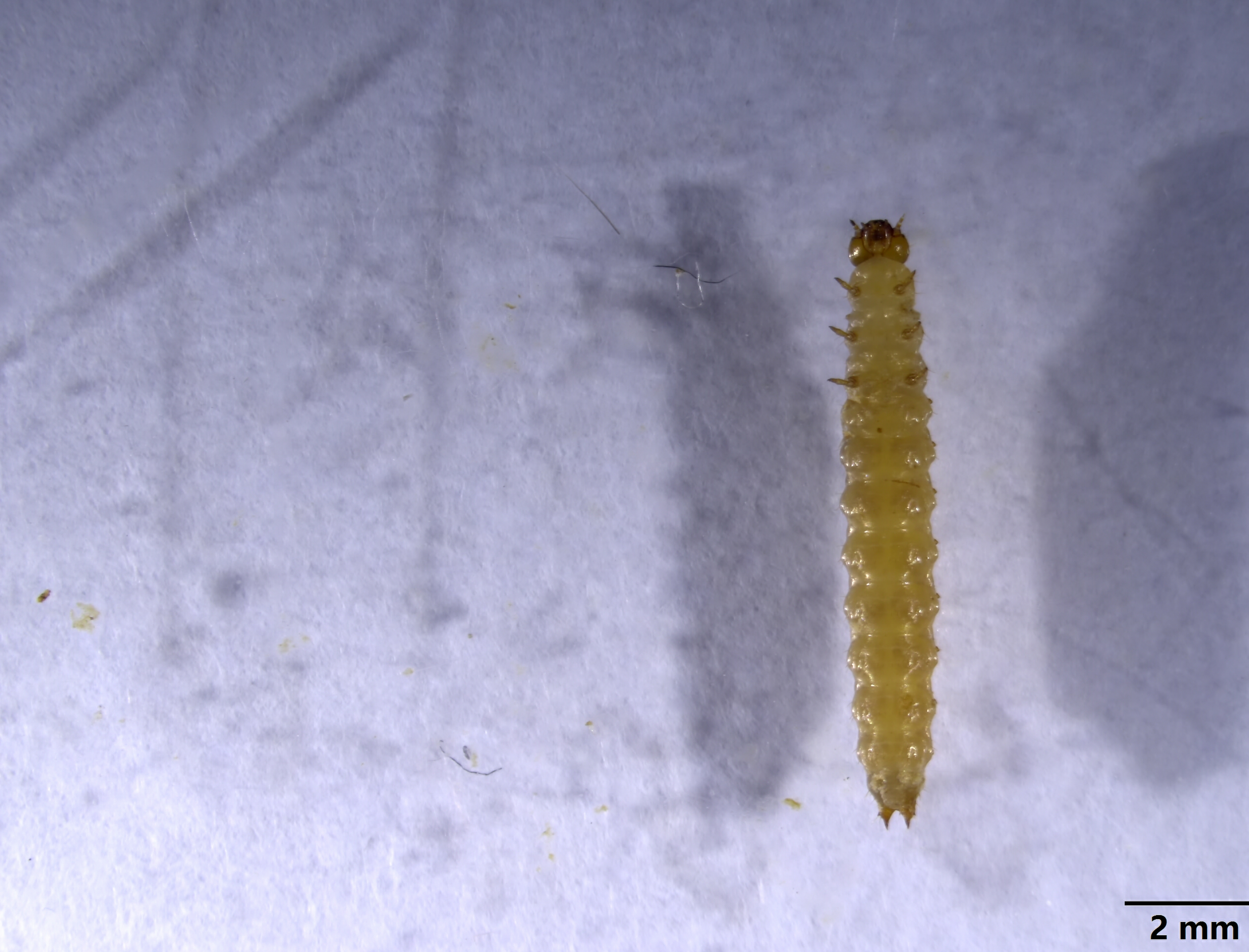

Supplement: Supplementary file 1 [file insects-17-00344-s001.zip › Experimental Data on Urophorus humeralis Nails/Figure/third-instar larvae/Ventral view.tif]
